# mTORC1 in Thymic Epithelial Cells Is Critical for Thymopoiesis, T-Cell Generation, and Temporal Control of γδT17 Development and TCRγ/δ Recombination

**DOI:** 10.1371/journal.pbio.1002370

**Published:** 2016-02-18

**Authors:** Hong-Xia Wang, Jinwook Shin, Shang Wang, Balachandra Gorentla, Xingguang Lin, Jimin Gao, Yu-Rong Qiu, Xiao-Ping Zhong

**Affiliations:** 1 Department of Pediatrics, Division of Allergy and Immunology, Duke University Medical Center, Durham, North Carolina, United States of America; 2 Laboratory Medicine Center, Nanfang Hospital, Southern Medical University, Guangzhou, Guangdong, China; 3 School of Laboratory Medicine, Wenzhou Medical University, Wenzhou, Zhejiang, China; 4 Department of Immunology, Duke University Medical Center, Durham, North Carolina, United States of America; 5 Hematologic Malignancies and Cellular Therapies Program, Duke Cancer Institute, Duke University Medical Center, Durham, North Carolina, United States of America; National Cancer Institute, UNITED STATES

## Abstract

Thymus is crucial for generation of a diverse repertoire of T cells essential for adaptive immunity. Although thymic epithelial cells (TECs) are crucial for thymopoiesis and T cell generation, how TEC development and function are controlled is poorly understood. We report here that mTOR complex 1 (mTORC1) in TECs plays critical roles in thymopoiesis and thymus function. Acute deletion of mTORC1 in adult mice caused severe thymic involution. TEC-specific deficiency of mTORC1 (mTORC1KO) impaired TEC maturation and function such as decreased expression of thymotropic chemokines, decreased medullary TEC to cortical TEC ratios, and altered thymic architecture, leading to severe thymic atrophy, reduced recruitment of early thymic progenitors, and impaired development of virtually all T-cell lineages. Strikingly, temporal control of IL-17-producing γδT (γδT17) cell differentiation and *TCRVγ/δ* recombination in fetal thymus is lost in mTORC1KO thymus, leading to elevated γδT17 differentiation and rearranging of fetal specific *TCRVγ/δ* in adulthood. Thus, mTORC1 is central for TEC development/function and establishment of thymic environment for proper T cell development, and modulating mTORC1 activity can be a strategy for preventing thymic involution/atrophy.

## Abstract 2

The thymus is essential for making T cells but undergoes age- or stress-associated atrophy. This study demonstrates that mTOR complex 1 in thymic epithelial cells is crucial for correct thymic architecture and the production of mature T cells.

## Author Summary

The thymus is the primary organ for T cell generation. Abnormal thymus function profoundly affects host immunity and numerous diseases. Thymopoiesis and thymus function rely on orchestrated interaction between multiple cell types representing different origins. Among them, thymic epithelial cells (TECs) are crucial for thymus development and maintenance and T cell generation. How TEC development and function are regulated is poorly understood. The mammalian/mechanistic target of rapamycin (mTOR), a serine/threonine kinase, signals with two complexes, mTORC1 and mTOC2, to control metabolism, growth, proliferation, and survival. Using a mouse model with mTORC1 selectively ablated in TECs, we demonstrate that mTORC1 in TECs plays critical roles in thymopoiesis and thymus function. Absence of mTORC1 results in impaired TEC maturation and function, altered thymic architecture, severe thymic atrophy, and impaired development of virtually all T-cell lineages. Moreover, it also causes increased generation of IL-17–producing γδT (γδT17) cells and fetal-specific γδT subsets in adult thymus, revealing that mTORC1 in TECs is central for temporal control of γδT17 differentiation and *TCRVγ/δ* recombination. Our results establish mTORC1 as a central regulator for TEC development/function and for the establishment of normal thymic environment for proper T cell development. We suggest modulating mTORC1 activity as a strategy for preventing thymic involution/atrophy.

## Introduction

The thymus is the primary organ for T cell development and generation of a diverse repertoire of T cells that are crucial for host defense but are also self-tolerated. Thymic epithelial cells (TECs) are essential for thymopoiesis and establish an environment that properly nurtures T cell development [1]. TECs include cortical and medullary subsets that reside in different localizations in the thymus and perform distinct functions. While cortical thymic epithelial cells (cTECs) are important for positive selection of conventional TCRα/β T (cαβT) cells, medullary thymic epithelial cells (mTECs) induce negative selection of highly self-reactive T cells and generation of regulatory T cells (Tregs) [2–5]. Interestingly, TECs are dynamically regulated by the cTEC to mTEC ratios being highest in the fetus and progressively lower as the mouse matures. In adult mice, mTECs substantially outnumber cTECs [6]. Although several transcription factors such as Foxn1 and Aire and receptors such as RANK, CD40, and LTβR are found important for TEC development/function [7–9], mechanisms that control thymopoiesis and mTEC/cTEC ratios are poorly understood.

During T cell ontogeny, early T cell progenitors (ETPs) enter into the thymus at the corticomedullary junction. Following initial migration toward the cortex, ETPs, which are Lin^−^CD4^−^CD8^−^ double negative (DN) cells that express CD44, cKit, and CD24 but not CD25, undergo maturation sequentially through the DN2, DN3, and DN4 stages [10]. TCRγδ T (γδT)-cells arise from these DN stages and can further differentiate into effector lineages such as IFNγ-producing γδT1 and IL-17-producing γδT17-cells within the thymus [11]. Interestingly, γδT17 differentiation occurs predominantly in the fetal thymus [12, 13]. Additionally, several *TCRVγ* (*Vγ5* and *Vγ6*) and *TCRVδ1* segments recombine only in the fetal thymus [14, 15]. Whether and how TECs may nurture a thymic environment to confer such temporal control of γδT17 differentiation and fetal-specific *TCRVγ/Vδ* usage has been unclear.

DN thymocytes uncommitted to the γδT fate but with in-frame rearranged *TCRβ* may overcome the developmental checkpoint between DN3 and DN4 to reach the CD4^+^CD8^+^double positive (DP) stage and adopt the αβT fate. Expression of a functional TCRα/β that recognizes self-peptide-major histocompatibility complex (MHC) complexes presented by cTECs triggers positive selection for maturation to the CD4^+^CD8^−^ or CD4^−^CD8^+^ single positive (SP) stage [16]. After positive selection, SP thymocytes migrate to the thymic medulla in a CCR7-dependent manner [17]. In the medulla, mTECs present promiscuously expressed tissue-specific antigens (TRAs) to SP thymocytes to trigger negative selection of cells that express TCR with high affinity to TRAs [18]. Small subsets of αβTCR-expressing thymocytes adopt Treg and NKT cell fates. Both negative selection and Tregs are critical for self-tolerance to prevent autoimmune diseases. Abnormal TEC development and function can cause severe consequences such as immunodeficiency or autoimmune diseases in both humans and animals, exemplified by deficiencies in Foxn1 or Aire [7, 19, 20].

The serine/threonine kinase mammalian/mechanistic target of rapamycin (mTOR) has the ability to integrate various environmental and intracellular stimuli and cues to control cell growth, proliferation, survival, autophagy, and metabolism. Mammalian/mechanistic target of rapamycin complex 1 (mTORC1), one of the two complexes, which contains a crucial and unique adaptor molecule Raptor, phosphorylates multiple substrates such as S6K1 and 4E-BP1 to promote protein, nucleic acid, and lipid synthesis, which is crucial for cell growth and proliferation [21]. mTOR is activated in thymocytes following TCR engagement via both PI3K-Akt and RasGRP1-Ras-Erk1/2 pathways [22] and intrinsically controls the development and/or function of *i*NKT cells, Tregs, and cαβT cells [23–28]. However, whether mTOR plays a role in TECs to extrinsically control T cell development is unknown. In this report, we demonstrate that mTORC1/Raptor signaling in TECs is crucial for thymopoiesis and proper generation of multiple T cell lineages. Deficiency of mTORC1/Raptor in TECs causes severe thymic atrophy, altered thymic structure, decreased mTEC/cTEC ratios, and severely reduced production of cαβT cells, Tregs, *i*NKT cells, and γδT cells correlated with decreased recruitment of ETPs in the thymus. Moreover, fetal thymus restricted γδT17 differentiation and *TCRVγ5/6Vδ1* recombination occur in adult thymus in the absence of mTORC1 in TECs, suggesting that TECs and thymic environment rather than hematopoietic stem cells confer temporal control of γδT cell development.

## Results

### Acute Deletion of mTORC1/Raptor in Adult Mice Caused Severe Thymic Atrophy

**Fig 1:**
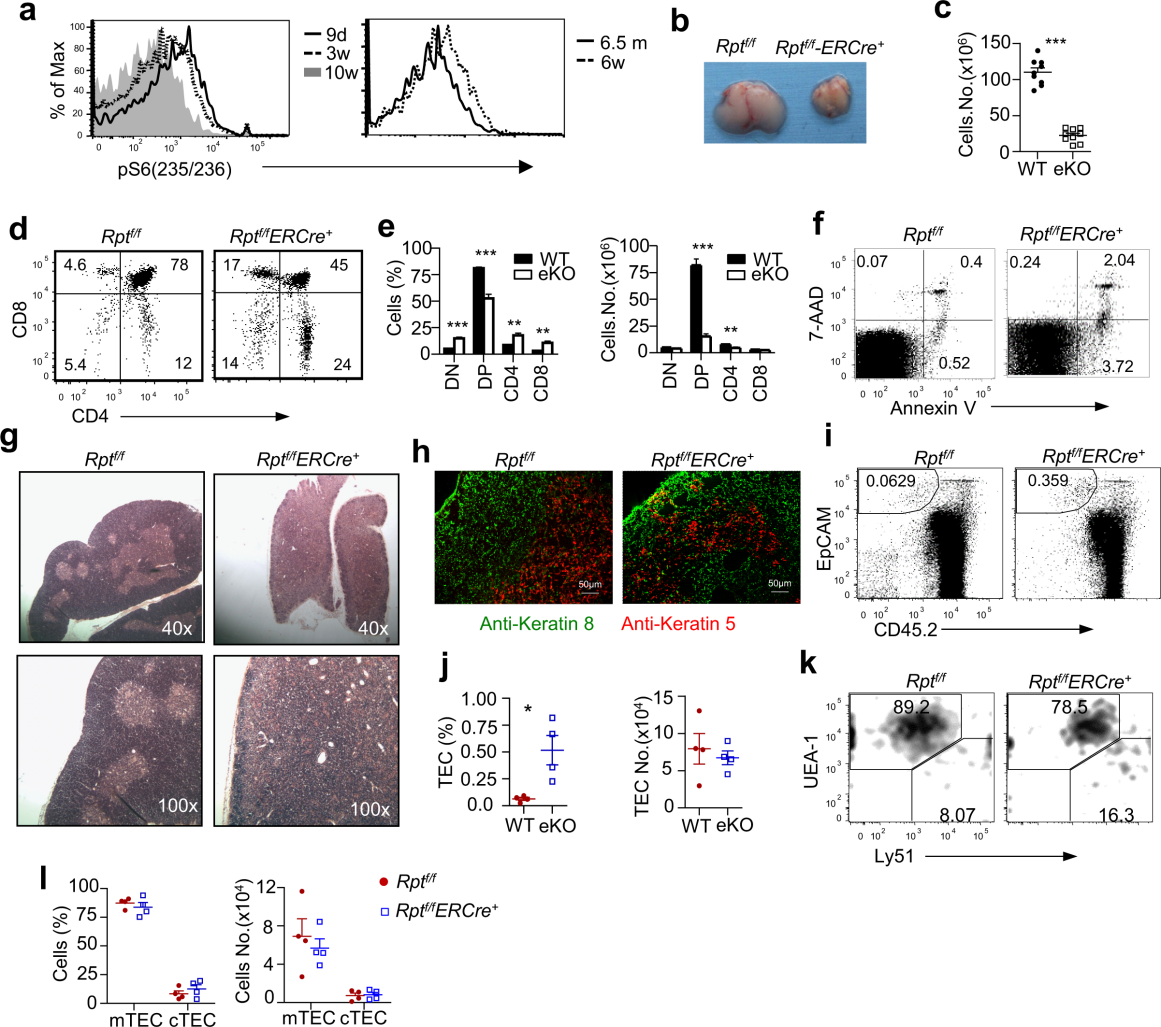
Acute deletion of mTORC1/Raptor induces thymic atrophy. **a.** Age-dependent decline of mTORC1 activity in TECs. Overlaid histograms show intracellular S6 phosphorylation in EpCAM^+^CD45^−^ TECs from C57BL6/J mice of indicated ages gated as described in S1A Fig. Data shown are representative of three experiments. **b.** Thymus size. For b to l: Thymic atrophy after acute systemic deletion of mTORC1. Six- to 8-wk-old *Rpt* ^*f/f*^ *-ERCre* and *Rpt* ^*f/f*^ mice were treated with tamoxifen on days 1, 2, and 5 and were euthanized on day 8. **c.** Total thymic cellularity. Bars represent mean ± standard error of the mean (SEM) (*n* = 9). Each circle or square represents one wild-type (WT) or knockout (KO) mouse, respectively. Numerical data for this figure and all other figures are in S1 Data. **d.** Thymocyte subsets. Representative dot-plots of CD4 and CD8 staining of thymocytes are shown. The gating strategy is shown in S1B Fig. **e.** Bar graphs represent mean ± SEM of percentages (left panel, *n* = 6) and absolute numbers (right panel, *n* = 5) of indicated thymocyte populations. **f.** Annexin-V and 7-AAD staining of DP thymocytes. The gating strategy is shown in S1C Fig. **g.** Hematoxylin and eosin (H&E) staining of thymic thin sections. **h.** Thymic cortex and medulla distribution revealed by immunofluorescence microcopy. Thymus cryosections were stained with primary rabbit anti-KRT5 and rat anti-KRT8 antibodies followed by secondary Rhodamine-labeled donkey anti-rabbit and fluorescein isothiocyanate (FITC)-labeled goat anti-rat antibodies. **i.** Dot-plot of TECs stained with anti-EpCAM and anti-CD45 antibodies. The gating strategy is shown in S1D Fig. **j.** Scatter plots represent mean ± SEM (*n* = 4) of EpCAM^+^CD45^−^ TEC percentages (left panel) and numbers (right panel). **k.** Distribution of UEA-1^+^Ly51^−^ mTECs and UEA-1^−^Ly51^+^ cTECs in gated CD45^−^EpCAM^+^ cells. The gating strategy is shown in S1D Fig. **l.** Percentages (left panel) and numbers (right panel) of mTECs and cTECs (*n* = 4). Data shown are representative of or calculated from at least three experiments. *p < 0.05; **p < 0.01; ***p < 0.001 determined by two-tailed Student’s *t* test.

We first examined S6 phosphorylation, an mTORC1/S6K1 dependent event, in TECs from mice aged at 9 d, 3 wk, and 10 wk. S6 phosphorylation was the strongest in TECs from 9-d-old mice but the lowest in cells from 10-wk-old adult mice (Fig 1A). Further comparison between 6-wk- and 6.5-mo-old mice revealed decreased S6 phosphorylation in TECs in aged mice (Fig 1A). Thus, mTORC1 activity in TECs appeared high in young mice but decreased with older age.

To determine the importance of mTORC1/Raptor signaling in thymus homeostasis, we examined 6–8-wk-old *Rptor* ^*f/f*^ *-Rosa26-ERCre (Rpt* ^*f/f*^ *-ERCre* or *eKO)* and control *Rptor* ^*f/f*^ (*Rpt* ^*f/f*^ or wild-type; WT) mice following tamoxifen injections on days 1, 2, and 5. On day 8, thymi in *Rpt* ^*f/f*^ *-ERCre* mice were much smaller than *Rpt* ^*f/f*^ mice (Fig 1B), accompanying a substantial decrease in total thymocyte numbers (Fig 1C). The percentage of CD4^+^CD8^+^DP thymocytes was decreased, but the percentages of CD4^−^CD8^−^DN, CD4SP, and CD8SP thymocytes were increased in *Rpt* ^*f/f*^ *-ERCre* mice (Fig 1D and 1E). The absolute number of DP thymocytes was severely decreased; CD4SP cell number was decreased by 50%; but DN and CD8SP thymocyte numbers were not obviously affected in tamoxifen-treated *Rpt* ^*f/f*^ *-ERCre* mice (Fig 1E). This was correlated with increased death in DP thymocytes (Fig 1F).

Hematoxylin and eosin (H&E) staining of thymus thin sections from tamoxifen-treated *Rpt* ^*f/f*^ *-ERCre* mice revealed abnormal thymus architecture: shrinkage of the cortex and increased presence of vacuous/cyst-like structures in medulla (Fig 1G), which were confirmed by immunofluorescence staining of cortex and medulla with anti-Keratin 8 (KRT8) and anti-Keratin 5 (KRT5) antibodies, respectively (Fig 1H). The shrinkage of cortex in *Rpt* ^*f/f*^ *-ERCre* mice was correlated with a sharp decrease in the number of DP thymocytes, which normally account for most cells in the cortex. In *Rpt* ^*f/f*^ *-ERCre* mice, although CD45^−^EpCAM^+^ TEC percentages were increased 6-fold (Fig 1I), their total numbers were similar to that of WT controls (Fig 1J). The predominance of UEA-1^+^Ly51^−^ mTECs over UEA-1^−^Ly51^+^ cTECs was not substantially disturbed in both percentages and numbers (Fig 1K and 1L). Thus, acute systemic mTORC1 deletion rapidly caused severe thymic atrophy, altered thymic architecture, and selectively reduced number of DP thymocytes.

### Crucial Role of mTORC1/Raptor in TECs for Proper Thymopoiesis

**Fig 2:**
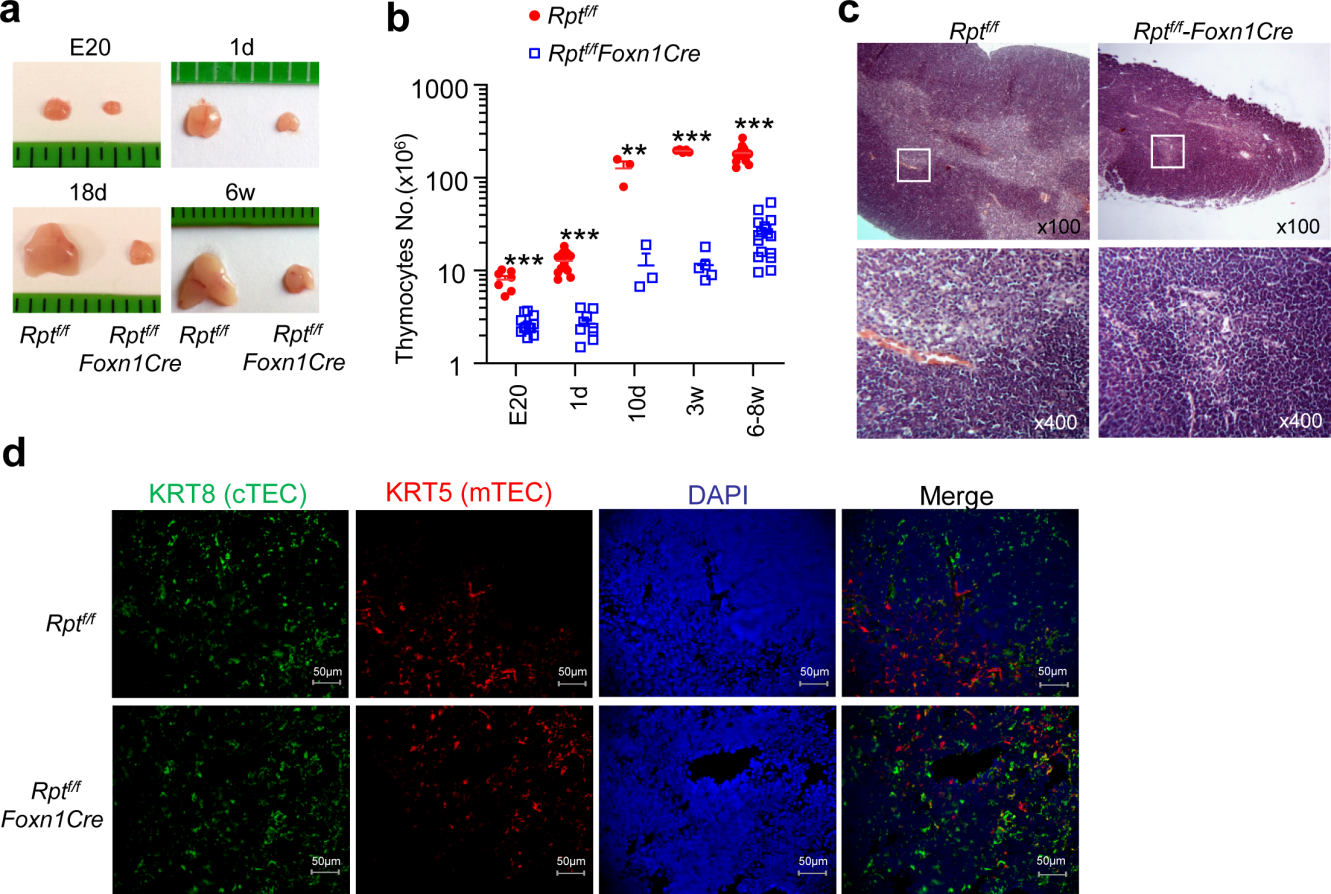
Thymic atrophy in mice with TEC-specific deletion of mTORC1/Raptor. *Rpt* ^*f/f*^ *-Foxn1Cre* and *Rpt* ^*f/f*^ mice of indicated ages were generated and examined. **a.** Thymus size. **b.** Total thymic cellularity. Each circle or square represents one WT and KO mouse, respectively. Bars represent mean ± SEM. **c.** H&E staining of thymus thin sections from a pair of 3-wk-old *Rpt* ^*f/f*^ *-Foxn1Cre* and *Rpt* ^*f/f*^ mice. **d.** Immunofluorescence analysis of thymus cryosections from a pair of 6-wk-old *Rpt* ^*f/f*^ *-Foxn1Cre* and *Rpt* ^*f/f*^ mice. Cryosections were stained similarly as Fig 1H. Data shown represent at least three experiments. **p < 0.01, ***p < 0.001 determined by two-tailed Student’s *t* test.

Although systemic deletion of mTORC1 caused severe thymic atrophy and decrease of DP thymocytes, T cell-specific ablation of mTORC1 in *Rpt* ^*f/f*^ *-LckCre* or *Rpt* ^*f/f*^ *-CD4Cre* mice did not cause obvious thymic atrophy or abnormal distribution of DN, DP, and SP populations in the thymus [28]. To test our hypothesis that mTORC1 might play a critical role in TECs for thymopoiesis and function, we generated and analyzed *Rpt* ^*f/f*^ *-Foxn1Cre* (knockout; KO) and *Rpt* ^*f/f*^ (WT) mice. *Foxn1Cre* mice contain an *IRES-Cre* cassette inserted into the 3’ untranslated region in the *Foxn1* locus to direct *Cre* expression starting on embryonic day 11.5 in TECs [29]. Strikingly, fetus (embryonic day 20, E20), newborn (1d), young (10 days, 10d; 18 days, 18d; 3 weeks, 3w), and adult (6–8 weeks, 6w) *Rpt* ^*f/f*^ *-Foxn1Cre* mice displayed apparent thymic atrophy (Fig 2A), accompanied by severely decreased total thymocyte numbers compared with R*pt* ^*f/f*^ controls (Fig 2B). Additionally, normal medullary and cortical structure was lost in *Rpt* ^*f/f*^ *-Foxn1Cre* thymus, which had a severely atrophied medulla (Fig 2C and 2D). Thus, mTORC1 in TECs was crucial for normal thymopoiesis.

### mTORC1/Raptor Signaling Ensures TEC Expansion, Maturation, and Proper Lineage Development

**Fig 3:**
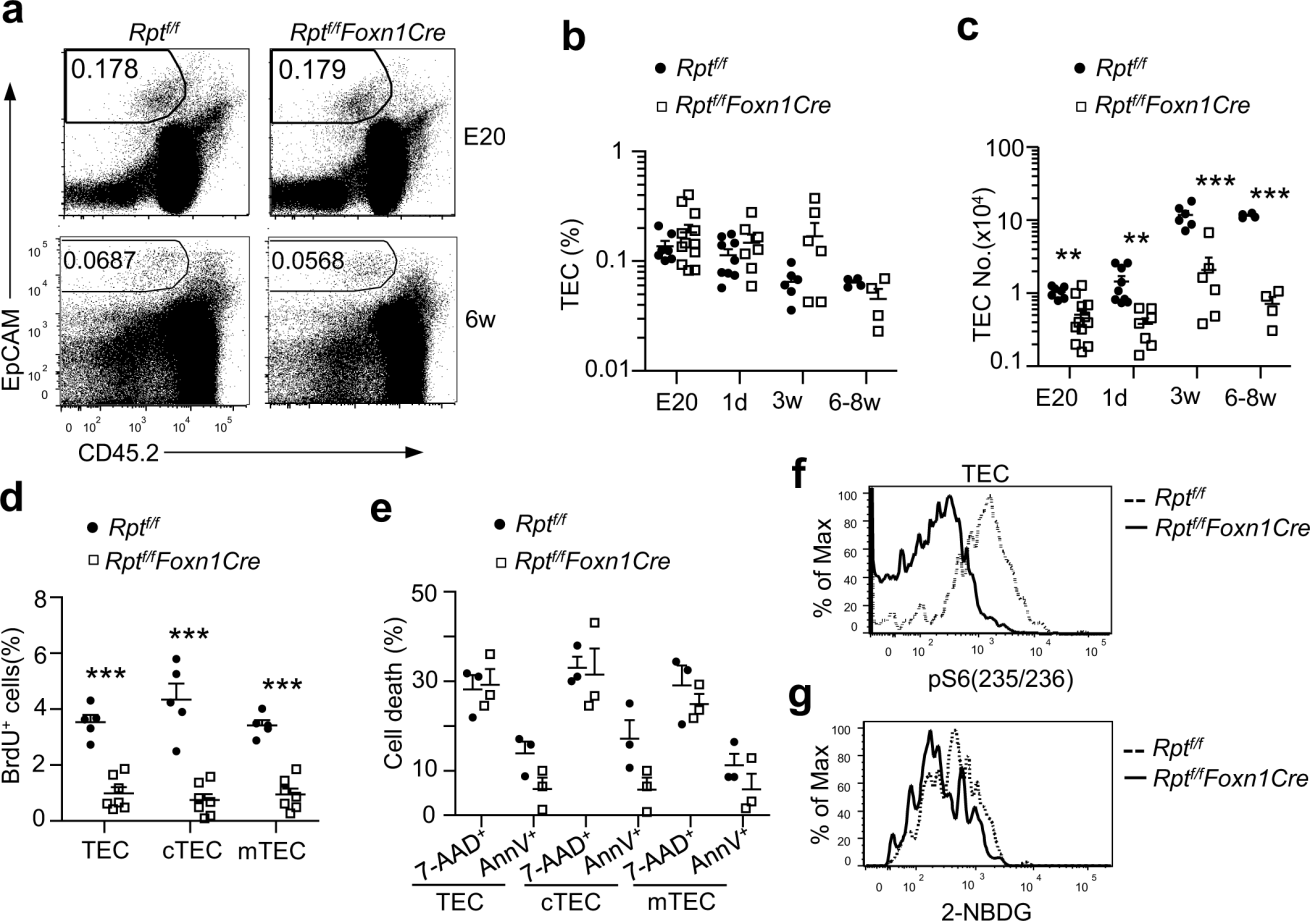
Severe decreases of TECs and impaired TEC expansion in the absence of mTORC1/Raptor. TECs from *Rpt* ^*f/f*^ *-Foxn1Cre* and *Rpt* ^*f/f*^ mice of indicated ages were stained with indicated antibodies or reagents and analyzed by flow cytometry. **a.** Representative dot-plots of CD45 and EpCAM expression on thymic cells. The gating strategy is shown in S2 Fig. **b.** Percentages of EpCAM^+^CD45^−^ TECs in thymus. **c.** Absolute numbers of TECs. **d.** Reduced TEC proliferation in Raptor-deficient mice. Three-wk-old mice were injected intraperitoneally (i. p.) with BrdU 3 times every 24 h and were euthanized for cell surface and intracellular staining of BrdU 14 h after last injection. **e.** Survival of TECs detected by 7AAD and annexin V staining. **f.** mTORC1 dependent S6 phosphorylation in TECs. Overlaid histograms show S6 phosphorylation in gated WT and *Rpt* ^*f/f*^ *-Foxn1Cre* EpCAM^+^CD45^-^ TECs. The gating strategy is the same as S2 Fig. **g.** Decreased glucose uptake in Raptor deficient TECs. The gating strategy is the same as S2 Fig. Data shown are representative or calculated from at least three experiments except E20, which was calculated from two experiments. Each circle or square represents one WT or *Rpt* ^*f/f*^ *-Foxn1Cre* mouse, respectively. *, *p* < 0.05; **, *p* < 0.01; ***, *p* < 0.001 determined by two-tail Student’s *t* test.

Severe thymic atrophy in *Rpt* ^*f/f*^ *-Foxn1Cre* mice suggested that mTORC1/Raptor signaling might be required for TEC development and/or function. Although TEC percentages from *Rpt* ^*f/f*^ *-Foxn1Cre* mice were not notably altered at indicated ages (Fig 3A and 3B), total TEC numbers were obviously decreased in *Rpt* ^*f/f*^ *-Foxn1Cre* thymi (Fig 3C). The magnitude of reduction was smaller at E20 but was progressively exacerbated as mice matured. Both Ly51^−^UEA-1^+^ mTEC and Ly51^+^UEA-1^−^ cTEC proliferation reflected by BrdU incorporation was decreased (Fig 3D), but their survival was not impaired in *Rpt* ^*f/f*^ *-Foxn1Cre* mice (Fig 3E). The impaired TEC proliferation was correlated with decreased S6 phosphorylation and, thus, reduced mTORC1 activity (Fig 3F) and decreased glucose uptake (Fig 3G). Thymic atrophy and reduced TECs in *Rpt* ^*f/f*^ *-Foxn1Cre* mice were not caused by expression of Cre protein itself in TECs, as thymi in *Rpt* ^*+/+*^ *-Foxn1Cre* mice and *Rpt* ^*f/f*^ mice were similar in sizes, total thymic cellularity, thymocyte subsets, and TEC numbers (S3A–S3H Fig). Together, these observations demonstrated that mTORC1 was critical for normal TEC development at least through promoting TEC expansion and glucose uptake.

**Fig 4:**
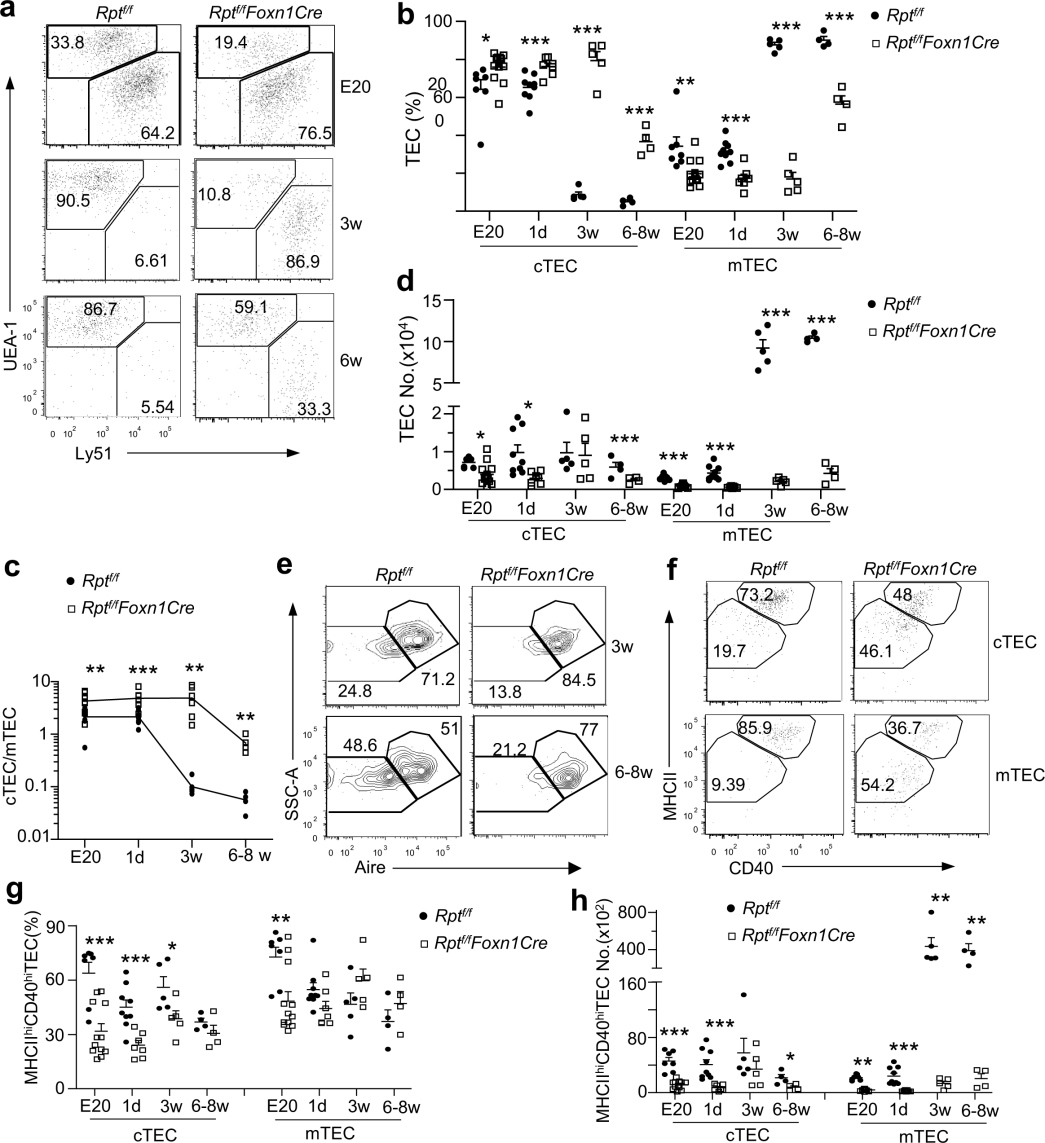
Decreased mTEC to cTEC ratios in the absence of mTORC1/Raptor. Thymic single cell suspension from *Rpt* ^*f/f*^ *-Foxn1Cre* and *Rpt* ^*f/f*^ mice of indicated ages were stained with anti-CD45, EpCAM, Ly5.1, UEA-1, MHC II, and CD40 antibodies and analyzed by flow cytometry. **a–d.** Altered m/cTEC ratios in *Rpt* ^*f/f*^ *-Foxn1Cre* mice. **a.** Representative plots of UEA-1 and Ly5.1 staining in gated EpCAM^+^CD45^−^ TECs. The gating strategy is shown in S4 Fig. **b.** Percentages of mTECs and cTECs. Bars indicate mean ± SEM. **c.** cTEC to mTEC ratios in fetal thymi and thymi of indicated ages. **d.** Absolute numbers of mTECs and cTECs. **e.** Aire expression in mTECs revealed by intracellular staining. The gating strategy is the same as S4 Fig. **f–h.** Impaired TEC maturation in *Rpt* ^*f/f*^ *-Foxn1Cre* mice. **f.** Representative dot-plots of MHC class II (MHCII) and CD40 expression on gated mTECs and cTECs from E20 thymi. Gating separates mTECs and cTECs into mature MHCII^hi^CD40^hi^ and immature MHCII^low^CD40^low^ populations. The gating strategy is the same as S4 Fig. **g.** Percentages of MHC-II^hi^CD40^hi^ mature TECs at the indicated ages. **h.** Cell numbers of MHC-II^hi^CD40^hi^ mature TECs at the indicated ages. Data shown are representative or calculated from at least four experiments except E20, which was calculated from two experiments. Each circle or square represents one *Rpt* ^*f/f*^ or *Rpt* ^*f/f*^ *-Foxn1Cre* mouse, respectively. *, *p* < 0.05; **, *p* < 0.01; ***, *p* < 0.001 determined by two-tail Student’s *t* test.

In WT mice, Ly51^+^UEA-1^−^ cTECs were about 2-fold more than Ly51^−^UEA-1^+^ mTECs in E20 and newborn thymi but accounted for about or less than 10% of total TECs after 3 wk of age (Fig 4A and 4B). In *Rpt* ^*f/f*^ *-Foxn1Cre* mice, cTEC percentages were 2–9-fold higher than WT controls from embryos to adulthood with the biggest difference at 3 wk of age (8.76 ± 1.40% WT versus 79.36 ± 4.75% KO), resulting in substantial decreases of mTEC/cTEC ratios after 3 wk of age (Fig 4C). Noticeably, due to severe decreases of total TECs, cTEC numbers were also decreased in *Rpt* ^*f/f*^ *-Foxn1Cre* mice throughout their life span except at 3 wk of age (Fig 4D). Expression of Aire, a transcription factor critical for mTEC maturation [20], was not decreased but rather slightly increased in *Rpt* ^*f/f*^ *-Foxn1Cre* mTECs (Fig 4E), ruling out decreased Aire expression as a causal factor of impaired TEC maturation/maintenance.

Both cTECs and mTECs can be defined into MHCII^low^CD40^low^ immature and MHC-II^hi^CD40^hi^ mature stages [30, 31]. From embryos to 3-wk-old mice, fewer *Rpt* ^*f/f*^ *-Foxn1Cre* cTECs reached mature stage than WT controls (Fig 4F and 4G). Such phenotype was not observed in adult mice. For mTECs, under-representation of mature stage was only observed in embryos but not after birth. The relatively unimpaired mTEC maturation was correlated with elevated Aire expression. However, due to a severe decrease of total TECs, mature mTECs and cTECs were considerably decreased throughout the life span of *Rpt* ^*f/f*^ *-Foxn1Cre* mice (Fig 4H).

Together, these observations demonstrated that mTORC1 is important for TEC expansion and efficient maturation and for establishing mTEC predominance over cTECs after adolescence.

### mTORC1/Raptor Deficiency in TECs Impaired Conventional αβT Cell Generation

**Fig 5:**
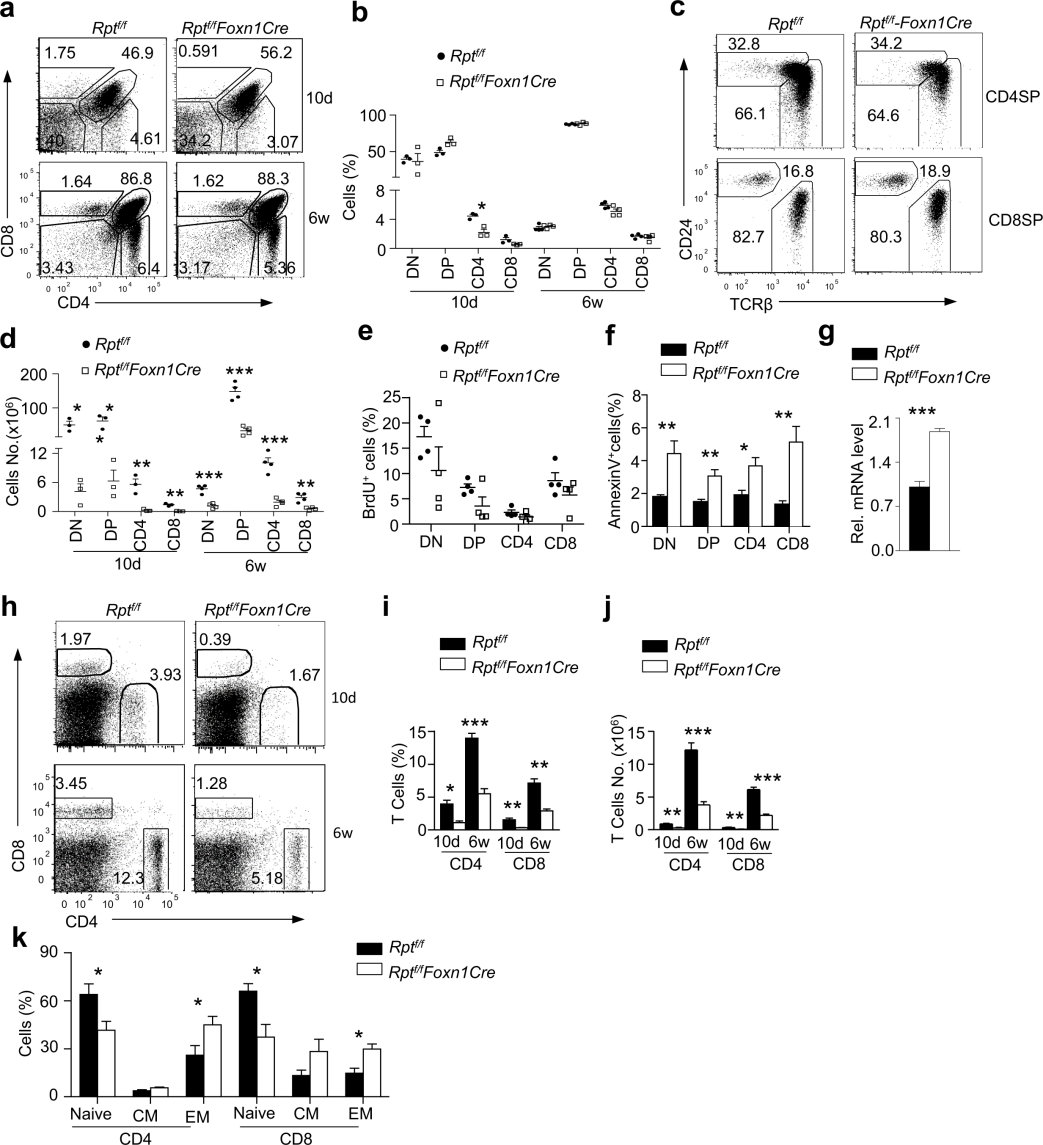
Impaired cαβT cell development in *Rpt* ^*f/f*^ *-Foxn1Cre* mice. **a.** CD4 and CD8 staining of thymocytes in 10-d- and 6-wk-old WT and *Rpt* ^*f/f*^ *-Foxn1Cre* mice. The gating strategy is shown in S5A Fig. **b.** Percentages of indicated thymocyte populations. Each circle or square represent one WT and *Rpt* ^*f/f*^ *-Foxn1Cre* mice respectively (10d, *n* = 3; 6w, *n* = 4). **c.** CD24 and TCRβ expression in CD4SP and CD8SP thymocytes from 6-wk old mice. **d.** Cell numbers of indicated thymocyte populations. **e.** BrdU incorporation in thymocytes. Mice were i. p. injected with 1 mg BrdU in 200 μl PBS for 4 h before euthanization for thymocyte staining. Scatter plot shows percentages of BrdU^+^ cells in indicated populations (*n* = 4). **f.** Increased death of thymocytes from *Rpt* ^*f/f*^ *-Foxn1Cre* mice. Bar graphs represent mean ± SEM of annexin V^+^ cells (*n* = 5). **g.** qPRC assessment of IL-7 mRNA in WT and *Rpt* ^*f/f*^ *-Foxn1Cre* TECs. **h.** Representative dot-plots of CD4 and CD8 staining in splenocytes. The gating strategy is shown in S5B Fig. **i.** Bar graphs represent mean ± SEM of CD4 and CD8 T cell (10d, *n* = 3; 6w *n* = 4). **j.** Bar graphs represent mean ± SEM of CD4 and CD8 T cell numbers (10d, *n* = 3; 6w *n* = 4). **k.** Increased effector/memory-like but decreased naïve T cells in 6–8-wk-old *Rpt* ^*f/f*^ *-Foxn1Cre* mice. Bar graphs represent mean ± SEM (*n* = 3). Data shown represent or are calculated from at least three experiments. *p < 0.05, **p < 0.01, ***p < 0.001 determined by two-tailed Student’s *t* test.

We further examined the impact of mTORC1 deficiency in TECs on T cell development. Percentages of DN, DP, CD4SP, and CD8SP thymocytes in *Rpt* ^*f/f*^ *-Foxn1Cre* were similar to WT controls at both 10-d and 6-wk of age (Fig 5A and 5B), except that CD4SP thymocyte percentage was decreased by 50% in *Rpt* ^*f/f*^ *-Foxn1Cre* thymus at 10 d. Within CD4SP and CD8SP cells, the ratios of TCRβ^+^CD24^−^ mature population were not obviously reduced (Fig 5C), suggesting that maturation of SP thymocytes was unhindered. Due to the drastic decrease of total thymic cellularity, the absolute numbers of all these populations were severely decreased in *Rpt* ^*f/f*^ *-Foxn1Cre* mice (Fig 5D). BrdU incorporation in DN, DP, and SP thymocytes was similar or only slightly decreased (Fig 5E), but annexin V^+^ apoptotic cells in these populations were increased (Fig 5F), suggesting that mTORC1 in TECs promotes thymocyte survival. IL-7, a pro-survival cytokine, was not decreased but actually increased at the mRNA level in *Rpt* ^*f/f*^ *-Foxn1Cre* TECs (Fig 5G), implying that mTORC1 may not control thymocyte survival via upregulating IL-7 transcription. However, due to the scarcity of TECs, we could not measure IL-7 protein in TECs and thus could not rule out that mTORC1 may promote thymocyte survival via increasing IL-7 translation. Nevertheless, our data demonstrated that mTORC1/Raptor deficiency in TECs led to increased thymocyte death and impaired αβT cell production without causing a developmental blockade at specific developmental checkpoints.

T cells generated in the thymus populate peripheral lymphoid organs to perform their functions. Both CD4^+^ and CD8^+^ cαβT cell percentages and numbers in the spleen were considerably decreased in *Rpt* ^*f/f*^ *-Foxn1Cre* mice (Fig 5H–5J), without obviously skewing TCRVβ usage (S6 Fig). There were noticeable increases of CD44^+^CD62L^−^ or CD44^+^CD62L^+^ effector/memory-like CD4 and CD8 T cells but decreases in CD62L^+^CD44^−^ naïve T cells in *Rpt* ^*f/f*^ *-Foxn1Cre* mice (Fig 5K), which was likely caused by lymphopenic proliferation. Thus, decreased T cell output from *Rpt* ^*f/f*^ *-Foxn1Cre* thymus resulted in T cell lymphopenia.

### Efficient nTreg Development Requires mTORC1/Raptor Signaling in TECs

**Fig 6:**
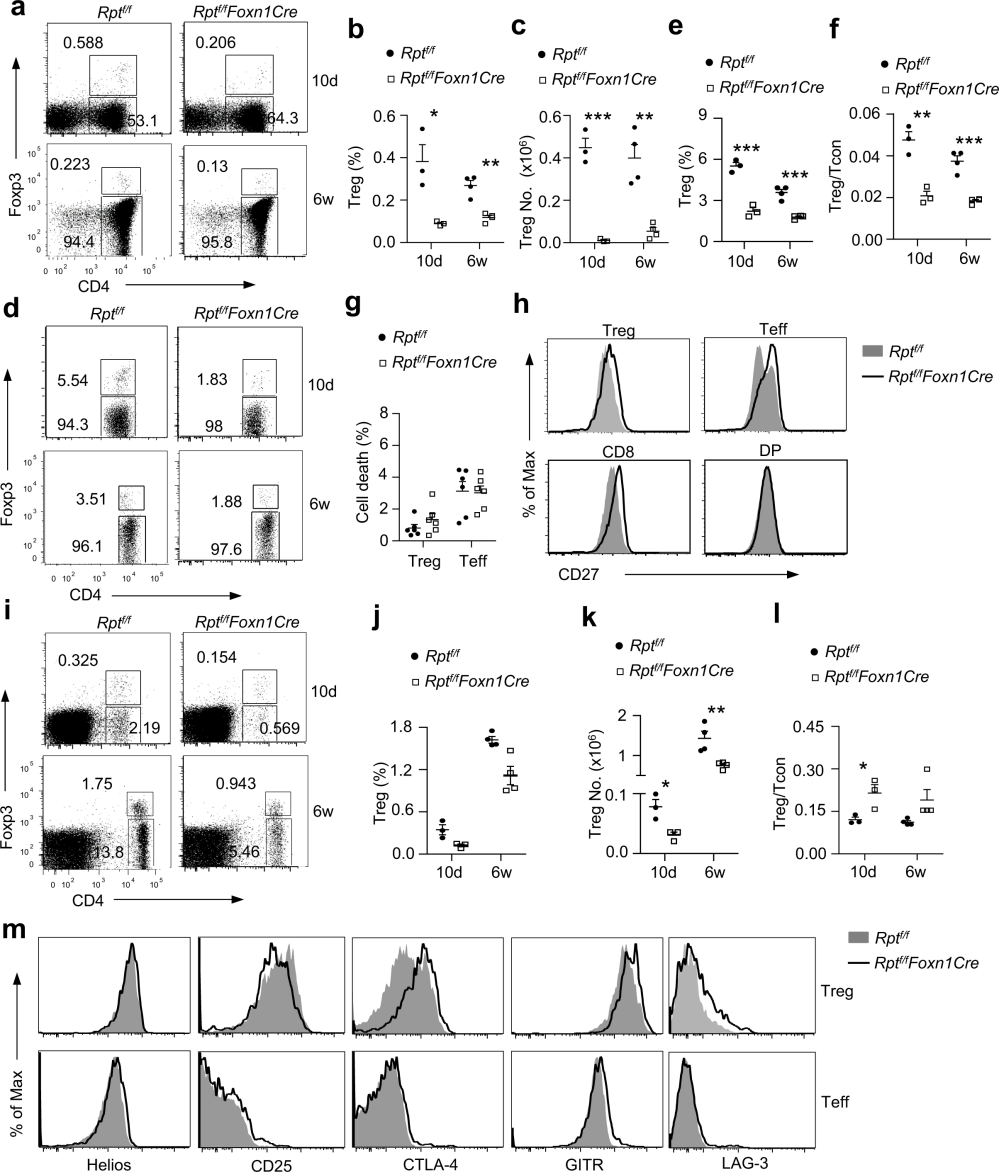
Impaired nTreg development in *Rpt* ^*f/f*^ *-Foxn1Cre* mice. Thymocytes (**a–f**) and splenocytes (**g–k**) from 10 d and 6 wk old *Rpt* ^*f/f*^ and *Rpt* ^*f/f*^ *-Foxn1Cre* mice were subjected to cell surface staining for the indicated molecules and intracellular staining for Foxp3 and Helios. **a.** Representative dot plots of CD4 and Foxp3 expression in live gated thymocytes. The gating strategy is shown in S7A Fig. **b.** Scatter plots show percentages of CD4^+^Foxp3^+^ Tregs in the thymus. Each circle or square represents one *Rpt* ^*f/f*^ or *Rpt* ^*f/f*^ *-Foxn1Cre* mouse. **c.** Total CD4^+^Foxp3^+^ Treg numbers in the thymus. **d.** Foxp3 staining in gated CD4^+^TCRβ^+^ thymocytes. The gating strategy is same as S7A Fig. **e.** Percentages of Foxp3^+^ cells within gated CD4^+^TCRβ^+^ thymocytes; **f.** Ratio of Foxp3^+^ to Foxp3^−^ cells within CD4^+^TCRβ^+^ thymocytes. **g.** Percentages of cell death of Foxp3^+^ and Foxp3^−^ cells within CD4^+^CD8^-^TCRβ^+^ thymocytes. **h.** Overlaid histograms show CD27 expression in Foxp3^+^CD4^+^TCRβ^+^, Foxp3^-^CD4^+^TCRβ^+^, CD4^−^CD8^+^TCRβ^+^, and CD4^+^CD8^+^ thymocytes. The gating strategy is shown in S7B Fig. **i.** Representative dot plots of CD4 and Foxp3 expression in live gated splenocytes. The gating strategy is shown in S7C Fig. **j.** Scatter plots show percentages of CD4^+^Foxp3^+^ Tregs in the spleen. Each circle or square represents one *Rpt* ^*f/f*^ or *Rpt* ^*f/f*^ *-Foxn1Cre* mouse. **k.** Total CD4^+^Foxp3^+^ Treg numbers in the spleen. **l.** Ratio of Foxp3^+^ to Foxp3^−^ cells within CD4^+^TCRβ^+^ splenocytes. **m.** Overlaid histograms show expression of indicated Treg signature molecules in Foxp3^+^CD4^+^ and Foxp3^−^CD4^+^ splenocytes. The gating strategy is the same as S7C Fig. Data shown are representative or calculated from at least three experiments (a–g, i–l) or two experiments (h, m). *, *p* < 0.05; **, *p* < 0.01; ***, *p* < 0.001 determined by tailed Student’s *t* test.

Recent studies have implicated mTECs for nTreg development [3, 4]. The severe reduction of mTECs in *Rpt* ^*f/f*^ *-Foxn1Cre* mice prompted us to examine whether nTreg development was jeopardized. As with CD4^+^Foxp3^−^ Teff, CD4^+^Foxp3^+^, nTreg percentages and numbers were substantially decreased in *Rpt* ^*f/f*^ *-Foxn1Cre* thymi at both 10 d and 6 wk of age (Fig 6A–6C). Moreover, nTreg percentages within CD4^+^TCRβ^+^ cells and the Treg/Teff ratios were more than 50% lower than those in WT controls (Fig 6D–6F), suggesting a more severely compromised nTreg development than Teff in *Rpt* ^*f/f*^ *-Foxn1Cre* mice. One potential mechanism for the severe nTreg developmental defect in *Rpt* ^*f/f*^ *-Foxn1Cre* mice could be the increased death of these cells. However, *Rpt* ^*f/f*^ *-Foxn1Cre* nTregs were not obviously prone to death when compared with control mice (Fig 6G). Expression of CD27, which promotes nTreg survival and generation [5], was not decreased but slightly increased in nTregs, Foxp3^-^CD4^+^CD8^-^ SP, and CD4^-^CD8^+^ SP thymocytes from *Rpt* ^*f/f*^ *-Foxn1Cre* mice (Fig 6H). Additional studies are needed to determine whether an abnormal CD27 costimulatory signal or other mechanisms contribute to severely compromised nTreg generation in *Rpt* ^*f/f*^ *-Foxn1Cre* mice.

In *Rpt* ^*f/f*^ *-Foxn1Cre* mice, splenic Treg percentages and numbers were also reduced within total splenocytes (Fig 6I–6K). However, Treg/Teff ratios were about 1.5-fold higher than WT controls (Fig 6L). These Foxp3^+^ Treg expressed nTreg marker Helios (Fig 6M), indicating that the relative enrichment of Tregs in the periphery of *Rpt* ^*f/f*^ *-Foxn1Cre* mice was likely caused by lymphopenia-induce proliferation because Treg expansion is superior to Teff under such condition. Tregs from *Rpt* ^*f/f*^ *-Foxn1Cre* mice expressed higher levels of several Treg signature molecules such as LAG3, CTLA-4, and GITR that facilitate their suppressive activity. Together, these observations indicated that mTORC1 in TECs plays important roles in nTreg differentiation.

### mTORC1/Raptor Signaling in TECs Is Critical for *i*NKT-Cell Development

**Fig 7:**
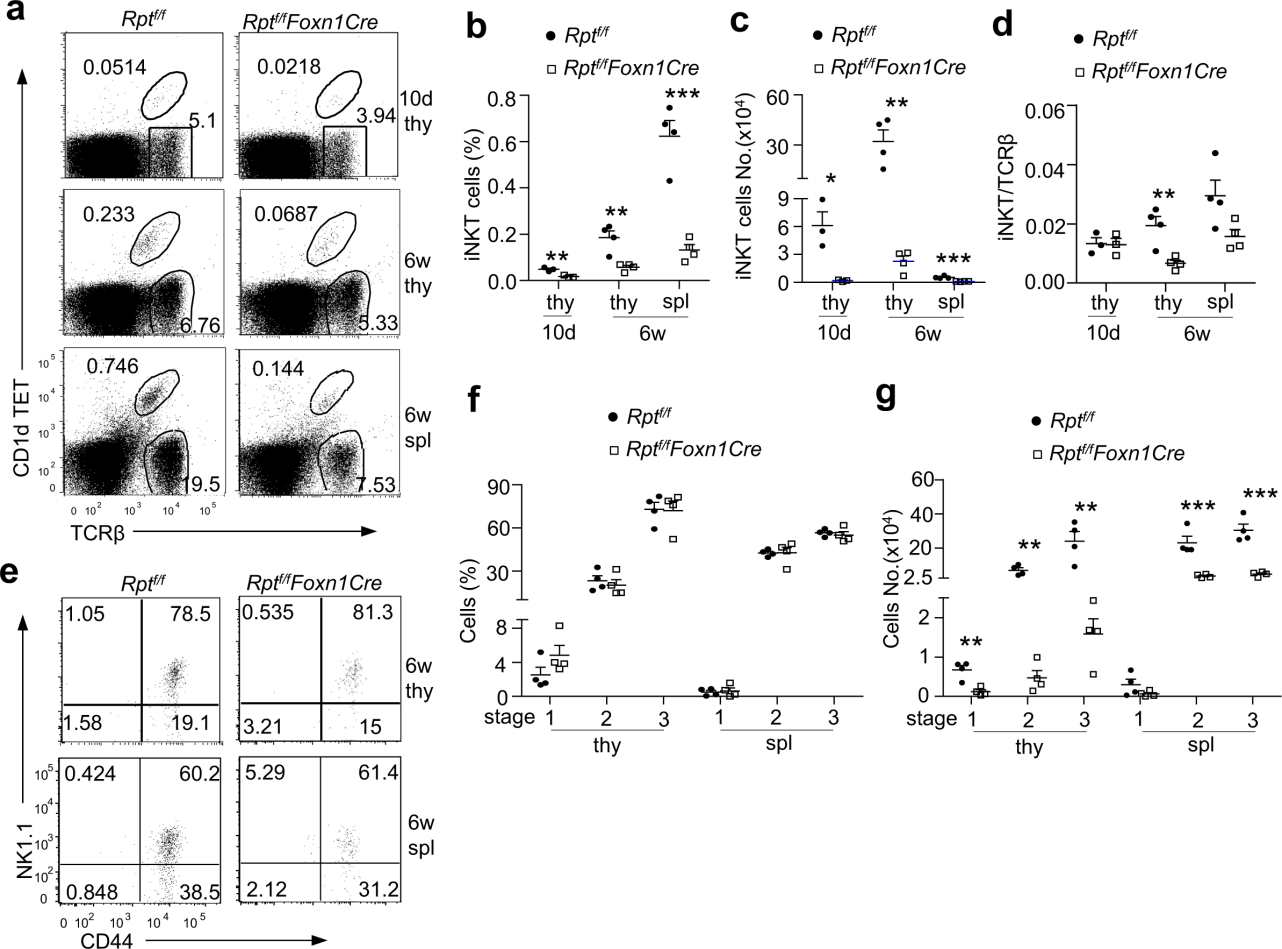
Severe *i*NKT cell developmental defect in *Rpt* ^*f/f*^ *-Foxn1Cre* mice. Thymocytes and splenocytes from 10-d- or 6-wk-old *Rpt* ^*f/f*^ (WT) and *Rpt* ^*f/f*^ *-Foxn1Cre* (KO) mice were stained with PBS57 loaded CD1d-Tetramer (CD1dTET), TCRβ, and other indicated molecules. **a.** CD1dTET and TCRβ staining of live-gated thymocytes. The gating strategy is shown in S8A Fig. **b.** Percentages of *i*NKT cells. Each circle and square represent one *Rpt* ^*f/f*^ and *Rpt* ^*f/f*^ *-Foxn1Cre* mice, respectively (10 d, *n* = 3; 6 wk, *n* = 4). Horizontal bars represent means and SEM. **c.** Absolute numbers of *i*NKT cells. Each circle and square represent one *Rpt* ^*f/f*^ and *Rpt* ^*f/f*^ *-Foxn1Cre* mice, respectively (10 d, *n* = 3; 6 wk, *n* = 4). Horizontal bars represent means and SEM. **d.** *i*NKT cells to cαβT cell ratios in individual mice (10 d, *n* = 3; 6 wk, *n* = 4). **e.** Representative dot plots show CD44 and NK1.1 expression in gated *i*NKT cells. The gating strategy is shown in S8B Fig. **f.** Percentages of indicated *i*NKT cell populations (*n* = 4). **g.** Absolute numbers of indicated *i*NKT cell populations (*n* = 4). Data shown are representative of or are calculated from at least three experiments. *, *p* < 0.05; **, *p* < 0.01; ***, *p* < 0.001 determined by tailed Student’s *t* test.

The invariant Vα14-Jα18 TCR-expressing NKT cells (*i*NKT) cells are also generated in the thymus. Unlike cαβT cells, they are positively selected after engagement of the iVα14TCR with self-lipid ligand-CD1d complex expressed on DP thymocytes [32]. The role of TECs in exogenously controlling *i*NKT cell development is largely unclear. The percentages of PBS57-loaded CD1d-Tetramer (CD1Dtet)^+^ TCRβ^+^ *i*NKT cells were decreased in the thymus of 10-d- and 6-wk-old *Rpt* ^*f/f*^ *-Foxn1Cre* mice (Fig 7A and 7B), with more drastic decreases of *i*NKT cell total numbers (Fig 7C). Moreover, although the ratio of *i*NKT to cαβT was comparable between *Rpt* ^*f/f*^ *-Foxn1Cre* and *Rpt* ^*f/f*^ control mice at 10 d of age, it decreased by 67% in adult *Rpt* ^*f/f*^ *-Foxn1Cre* thymus (Fig 7D), suggesting more defective *i*NKT generation than cαβT cells in adult mice. Although IL-15 expressed by mTECs promotes late stage *i*NKT cell development [33] and the mTEC number was severely decreased in *Rpt* ^*f/f*^ *-Foxn1Cre* thymus, no obvious late stage *i*NKT developmental blockade was observed. The relative percentages of stages 1 (CD24^−^CD44^−^NK1.1^−^), 2 (CD24^−^CD44^+^NK1.1^−^), and 3 (CD24^−^CD44^+^NK1.1^+^) *i*NKT cells in these mice were similar to WT controls (Fig 7E and 7F), although their absolute numbers were markedly decreased (Fig 7G). Just like in the thymus, *i*NKT cell percentages and numbers were obviously decreased in the spleen (Fig 7A–7C). Together, these observations indicate that *i*NKT cell generation is dependent on mTORC1 signaling in TECs.

### mTORC1/Raptor in TECs Ensures Temporal Control of TCRVγ Usage and γδT17 Differentiation

**Fig 8:**
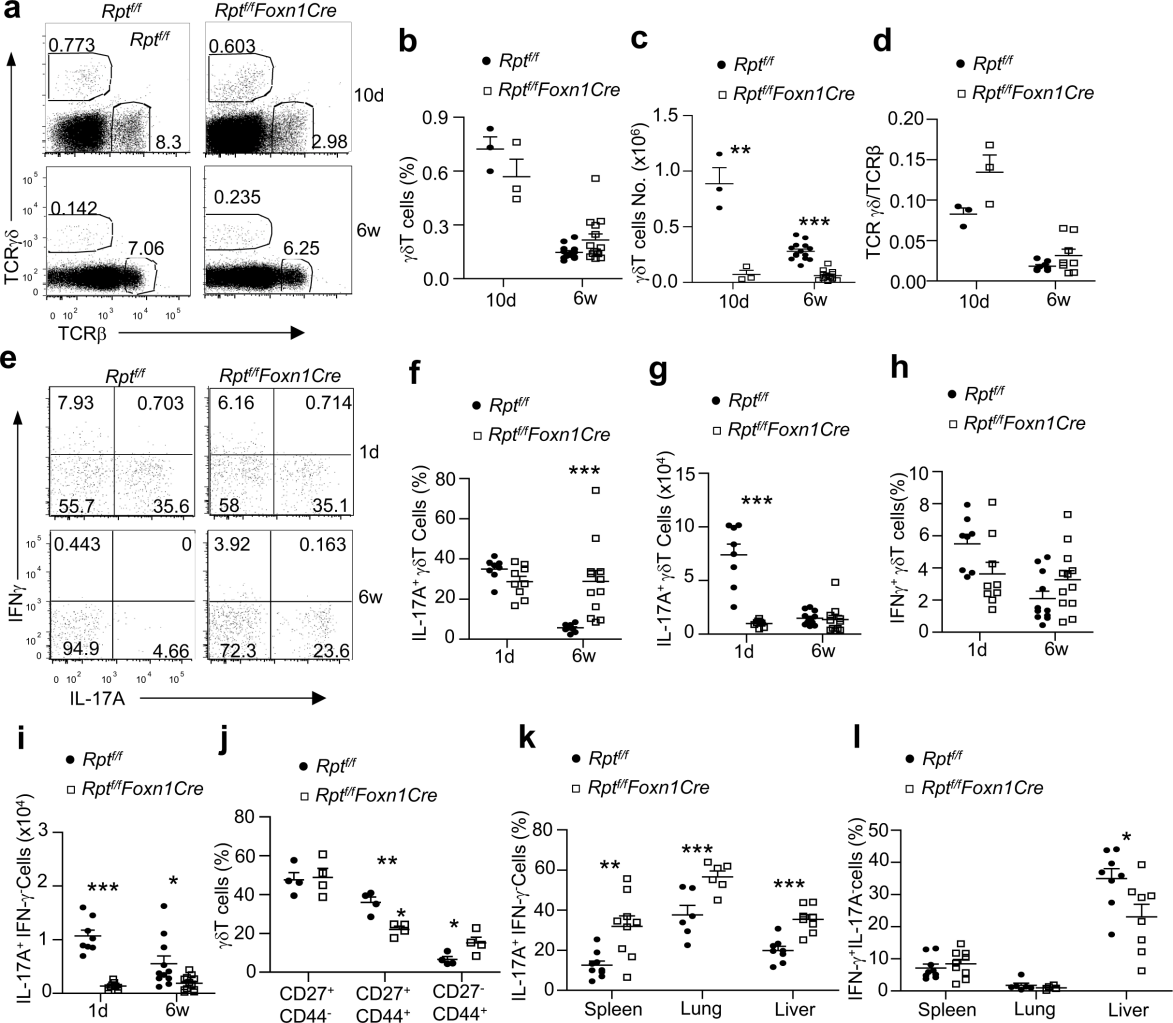
Enhanced γδT17 lineage differentiation in adult mTORC1/Raptor deficient mice. **a.** Representative dot plots showing TCRγδ and TCRβ expression in 10-d- and 6-wk-old WT and *Rpt* ^*f/f*^ *-Foxn1Cre* thymocytes. The gating strategy is shown in S9A Fig. **b.** Scatter plots show γδT percentages in 10-d- and 6-wk-old WT and *Rpt* ^*f/f*^ *-Foxn1Cre* thymus (10 d, *n* = 3; 6 wk, *n* = 4). Each circle and square represents one WT and *Rpt* ^*f/f*^ *-Foxn1Cre* mouse, respectively. Bars represent mean ± SEM. **c.** Scatter plots show γδT numbers in 10-d- and 6-wk-old WT and *Rpt* ^*f/f*^ *-Foxn1Cre* thymus (10 d, *n* = 3; 6 wk, *n* = 4). Each circle and square represents one WT and *Rpt* ^*f/f*^ *-Foxn1Cre* mouse, respectively. Bars represent mean ± SEM. **d.** γδT/cαβT ratios in the thymus. **e–g.** Enhanced γδT17 differentiation in *Rpt* ^*f/f*^ *-Foxn1Cre* mice. Thymocytes from newborn (1 d) and 6-wk-old WT and *Rpt* ^*f/f*^ *-Foxn1Cre* mice were stimulated with phorbol myristate acetate (PMA) plus ionomycin in the present of GolgiPlug for 4 h. Dot plots show intracellular IFNγ and IL-17A staining in gated TCRγδ^+^CD3^+^ cells (e). The gating strategy is shown in S9B Fig. Scatter plots represent mean ± SEM of γδT17-cells (f, percentages; g, numbers) and γδT1-cells (h, percentages; i, numbers). **j.** Altered γδT cell subsets in *Rpt* ^*f/f*^ *-Foxn1Cre* mice. Scatter plots show percentages of the CD27^+^CD44^−^, CD27^+^CD44^+^, and CD27^−^CD44^+^ populations of thymic γδT cells from 6-wk-old WT and *Rpt* ^*f/f*^ *-Foxn1Cre* thymus. Each circle and square represents one WT and *Rpt* ^*f/f*^ *-Foxn1Cre* mouse, respectively. Bars represent mean ± SEM. **k,l.** Increased γδT17 cells in peripheral organs in *Rpt* ^*f/f*^ *-Foxn1Cre* mice. Splenocytes, lung, and liver mononuclear cells from 6–8-wk-old mice were stimulated and analyzed similarly as in e–g. Scatter plots represent mean ± SEM of γδT17 cells (k) and γδT1 cells (l). Data shown represent or are calculated from at least three experiments. *, *p* < 0.05; **, *p* < 0.01; ***, *p* < 0.001 determined by two-tailed Student’s *t* test.

Unlike cαβT cells, most γδT cells develop independent of the MHC-mediated antigen presentation by TECs [34, 35]. Although γδT cell percentages in *Rpt* ^*f/f*^ *-Foxn1Cre* thymi were not obviously altered at 10 d and 6 wk of age (Fig 8A and 8B), total γδT cell numbers were substantially decreased (Fig 8C). However, unlike nTreg and *i*NKT cells, γδT to cαβT ratios were not decrease or even slightly increased in *Rpt* ^*f/f*^ *-Foxn1Cre* mice (Fig 8D), suggesting that γδT cell generation was dependent on mTORC1 in TECs but appeared less severely affected than nTregs and *i*NKT cells.

Considerable numbers of γδT cells are programmed to differentiate to distinct effector lineages within the thymus [34–36]. γδT17 cells are developed mostly in fetal thymus [12, 13]. Similarly, *TCRVγ5*, *Vγ6*, and *Vδ1* only recombine in fetal thymus [11, 14]. Mechanisms that enforce such temporal controls are unknown. In WT thymus, γδT17 cells accounted for about 40% and 5% of total γδT cells at birth and 6 wk of age, respectively. In *Rpt* ^*f/f*^ *-Foxn1Cre* mice, γδT17 percentages did not differ greatly at birth but increased 5-fold in adults compared to WT controls (Fig 8E and 8F). Although total thymic γδT17 numbers were decreased in newborn *Rpt* ^*f/f*^ *-Foxn1Cre* thymi, they were similar to WT controls when 6 wk old (Fig 8G). Because adult *Rpt* ^*f/f*^ *-Foxn1Cre* thymi were much smaller than WT controls, similar total γδT17 cell numbers in these mice suggested that γδT17 generation was greatly favored in adult *Rpt* ^*f/f*^ *-Foxn1Cre* thymi, although thymic γδT1 ratio was not altered (Fig 8E and 8H) and γδT1 numbers were obviously decreased in *Rpt* ^*f/f*^ *-Foxn1Cre* mice (Fig 8I). Additionally, CD44^+^CD27^−^ γδT cells, which were enriched with γδT17 [13], were also increased in *Rpt* ^*f/f*^ *-Foxn1Cre* thymi (Fig 8J). Concordantly, γδT17 but not γδT1 percentages in splenic, lung, and liver γδT cells were also increased in *Rpt* ^*f/f*^ *-Foxn1Cre* mice (Fig 8K and 8L). To determine whether Cre protein expression in TECs was sufficient to cause enhanced γδT17 generation, we compared γδT1/17 differentiation in *Rpt* ^*+/+*^ *-Foxn1Cre* mice and *Rpt* ^*+/+*^ mice. As shown in S10A–S10D Fig, *Rpt* ^*+/+*^ *-Foxn1Cre* thymic γδT cells were not obviously different from *Rpt* ^*+/+*^ thymic γδT cells in percentages and numbers as well as in γδT1 and γδT17 percentages, suggesting that Cre expression in TECs per se was not able to cause the abnormalities in *Rpt* ^*f/f*^ *-Foxn1Cre* mice. Together, these observations revealed that mTORC1/Raptor in TECs prevented γδT17 generation in the adult thymus.

**Fig 9:**
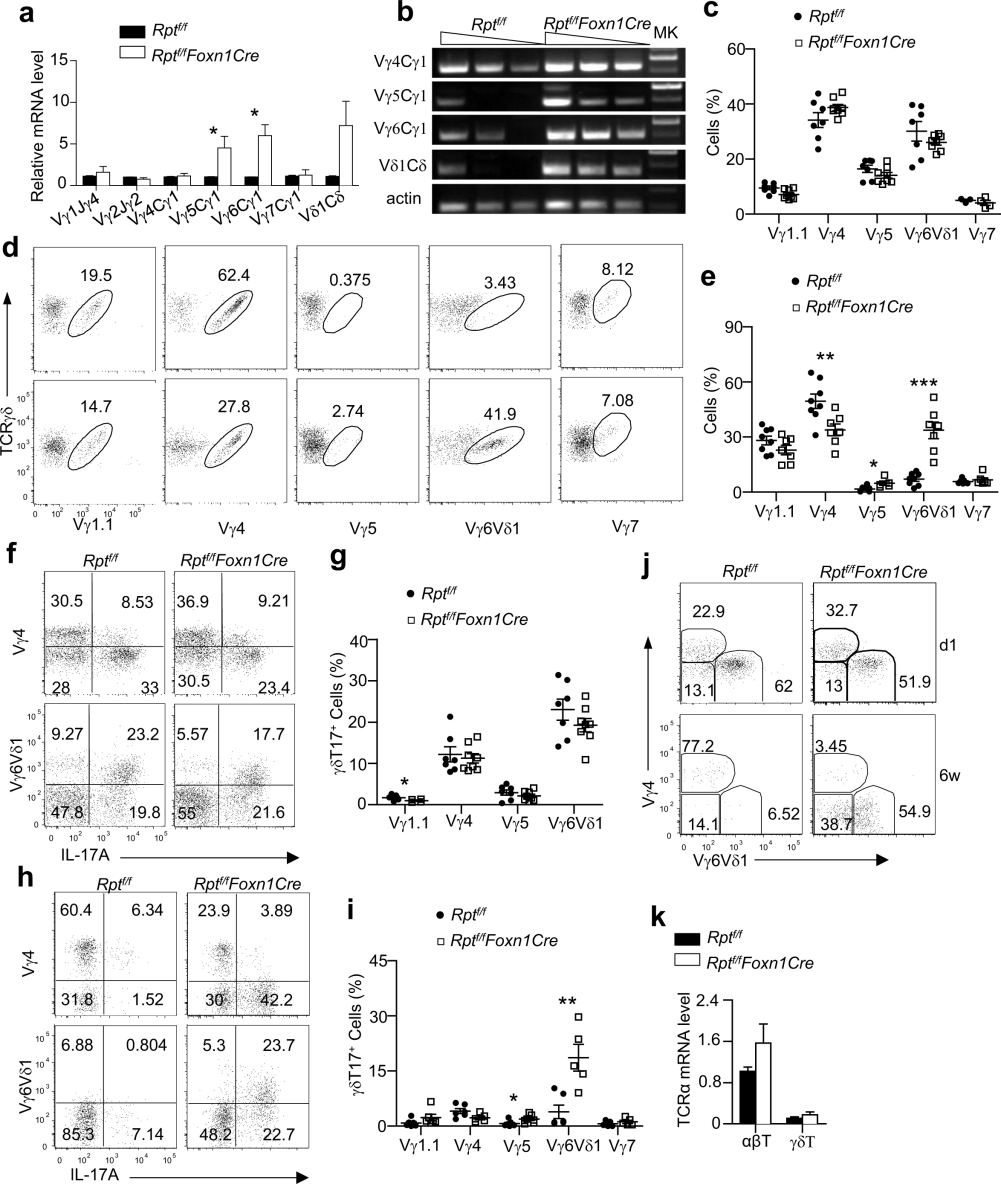
Impaired temporal control of fetal-specific TCRVγ/δ recombination and Vγ6Vδ1^+^ γδT17 differentiation in mTORC1/Raptor-deficient mice. Thymocytes from newborn or 6–10-wk-old adult *Rpt* ^*f/f*^ and *Rpt* ^*f/f*^ *-Foxn1Cre* mice were used for sorting γδT cells (a,b,k) and αβT cells (k), for directly assessing Vγ usages of γδT cells after cell surface staining (c–e), or for assessing Vγ usage of γδT17 cells after PMA plus ionomycin stimulation for 4 h (f–j). **a,b.** *Vγ1-Jγ4*, *Bγ2-Jγ2*, *Vγ4-Cγ1*, *Vγ5-Cγ1*, *Vγ6-Cγ1*, *Vγ7-Cγ1*, and *Vδ1-Cδ* mRNAs in sorted thymic γδT cells from 2-mo-old mice were quantified by real-time qPCR (a) and semiquantitative PCR (b). Data shown are mean ± SEM calculated from three experiments (a) or are representative of three experiments (b). Sorting strategy is shown in S11A Fig. **c.** TCRVγ distribution in newborn thymic γδT cells. **d,e.** TCRVγ distribution in adult thymic γδT cells. Dot plots show TCRγδ and indicated Vγ staining in gated TCRγδ^+^ cells (d). Scatter graphs show individual Vγ subsets within γδT cells. Bars represent mean ± SEM (e). The gating strategy is shown in S11B Fig. **f.** Representative dot plots show IL-17A and Vγ4 or Vγ6Vδ1 expression in gated newborn thymic γδT cells after PMA plus ionomycin stimulation for 4–5 h in the presence of GolgiPlug. Data shown represent at least three experiments. The gating strategy is shown in S11C Fig. **g.** TCRVγ distribution of thymic γδT17 cells in newborn mice. Scatter plots represent mean ± SEM of Vγ subsets of γδT17 cells. Data shown represent or are calculated from three experiments. **h.** Representative dot plots show IL-17A and Vγ4 or Vγ6Vδ1 expression in gated thymic γδT cells from adult mice after PMA plus ionomycin stimulation for 4 h in the presence of GolgiPlug. Data shown represent at least three experiments. The gating strategy is shown in S11D Fig. **i.** TCRVγ distribution of thymic γδT17 cells in adult mice. This is the same as in Fig 9G, except 6–10-wk-old mice were tested and the Vγ7^+^ subset was included. **j.** Representative dot plots show Vγ4 and Vγ6Vδ1 staining of gated γδT17 cells from newborn and adult thymi. The gating strategy is shown in S11E Fig. **k.** TCRα mRNA levels in sorted γδT and αβT cells from adult thymi detected by real-time qPCR. Data shown represent or are calculated from at least three experiments. *, *p* < 0.05; **, *p* < 0.01; ***, *p* < 0.001 determined by two-tailed Student’s *t* test.

Coinciding with the loss of temporal control of γδT17 differentiation, *Vγ5*, *Vγ*6, and *Vδ1* recombination, which occurs only in fetal thymi in WT mice, occurred at high levels in adult *Rpt* ^*f/f*^ *-Foxn1Cre* thymi (Fig 9A and 9B). Although γδT subsets defined by TCRVγ usages were similar in newborn WT and *Rpt* ^*f/f*^ *-Foxn1Cre* thymi (Fig 9C, S12 Fig), they were obviously different in adult thymi (Fig 9D and 9E). Both Vγ6Vδ1^+^ and Vγ5^+^ subsets were increased, but Vγ4^+^ subsets were decreased in relative ratios in adult *Rpt* ^*f/f*^ *-Foxn1Cre* thymi compared with WT controls; thus, mTORC1/Raptor in TECs enforced strict restriction of fetal-specific *Vγ5/6/Vδ1* recombination.

In newborn thymi, we observed similarly high percentages of γδT17 cells that were either Vγ4^+^ or Vγ6^+^, which accounted for most of γδT17 cells in a normal fetal thymus [12], in both WT and *Rpt* ^*f/f*^ *-Foxn1Cre* mice (Fig 9F and 9G). There were no obvious differences in γδT17 percentages in Vγ1^+^, Vγ4^+^, Vγ5^+^, and Vγ6Vδ1^+^ populations of γδT cells between newborn WT and *Rpt* ^*f/f*^ *-Foxn1Cre* thymi. However, γδT cells in adult *Rpt* ^*f/f*^ *-Foxn1Cre* thymi showed substantially increased Vγ6Vδ1^+^ and Vγ5^+^ γδT17 cells (Fig 9H and 9I). Most newborn γδT17 cells were either Vγ4^+^ or Vγ6Vδ1^+^ in both WT and *Rpt* ^*f/f*^ *-Foxn1Cre* mice. However, Vγ6Vδ1^+^ γδT cells accounted for the majority of γδT17 cells in *Rpt* ^*f/f*^ *-Foxn1Cre* thyme, and this population of γδT17 cells was 4-fold greater in *Rpt* ^*f/f*^ *-Foxn1Cre* thymi than in WT control (Fig 9J). A significant portion of γδT17 cells in adult *Rpt* ^*f/f*^ *-Foxn1Cre* thymi was Vγ4^−^Vγ6Vδ1^−^, which could be other Vγ subsets or poorly stained Vγ6Vδ1^+^ cells. However, they were unlikely αβT cells because they were TCRβ^−^. Moreover, γδT cells sorted from adult WT and *Rpt* ^*f/f*^ *-Foxn1Cre* thymi expressed similarly low levels of *TCRα* compared with αβT cells (Fig 9K).

**Fig 10:**
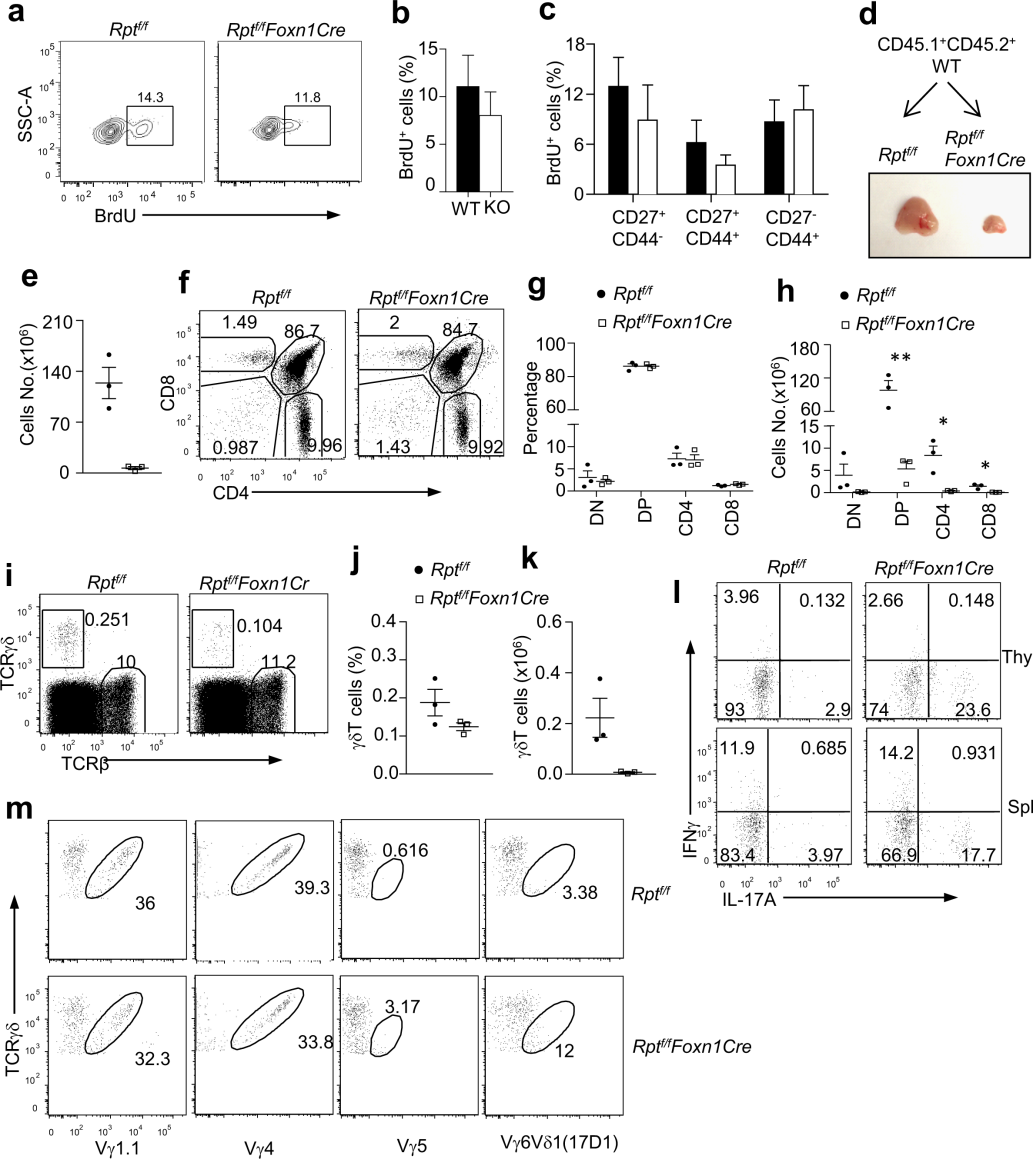
Adult *Rpt* ^*f/f*^ *-FoxnCre* thymi support γδT17 and Vγ6Vδ1 γδT cell generation from adult bone marrow. **a–c.** BrdU incorporation in thymic γδT cells. Three-week-old mice were i. p. injected with 1 mg BrdU in 200 μl PBS for 4 h before euthanization for thymocyte staining. Scatter plot shows percentages of BrdU^+^ cells in indicated populations (*n* = 4). **a.** Representative dot plots showing BrdU incorporation in gated γδT cells. The gating strategy is shown in S13A Fig. **b.** Bar graphs represent mean ± SEM of percentages of BrdU^+^ γδT cells. **c.** Bar graphs represent mean ± SEM of percentages of BrdU^+^ cells in the indicated γδT subsets. **d–m.** Lethally irradiated *Rpt* ^*f/f*^ *-FoxnCre* and *Rpt* ^*f/f*^ mice were reconstituted with T cell-depleted bone marrow cells from CD45.1^+^CD45.2^+^ WT mice. Five to six weeks after reconstitution, thymocytes and splenocytes from recipient mice were collected for fluorescence-activated cell sorting (FACS) analysis before or after PMA plus ionomycin stimulation. **d.** Thymus from recipient mice. **e.** Total thymic cellularity. **f.** Thymic subsets based on CD4 and CD8 staining. Representative dot plots of live-gated CD45.1^+^ thymocytes are shown. The gating strategy is shown in S13B Fig. **g.** Scatter plot shows percentages of individual thymocyte subsets. **h.** Cell numbers of CD45.1^+^ donor-derived thymic subsets. **i.** Representative dot plots show TCRβ and TCRγδ staining of live gated CD45.1^+^ donor-derived thymocytes. The gating strategy is the same as in S13C Fig. **j.** CD45.1^+^ donor-derived γδT cell percentages in thymi. **k.** CD45.1^+^ donor-derived γδT cell numbers in thymi. **l.** Representative dot plots showing IL-17A and IFNγ staining in gated CD45.1^+^ donor-derived thymic and splenic γδT cells. The gating strategy is same as S13C Fig. **m.** TCRVγ staining in gated CD45.1^+^ donor-derived γδT cells. The gating strategy is shown in S13D Fig. Data shown represent or are calculated from three experiments. Together, our data demonstrated that mTORC1/Raptor in TECs fostered a thymic environment that enforced temporal control on γδT17 differentiation and might play an important role for strict restriction of fetal-specific *Vγ5/6/Vδ1* recombination.

One possible reason for increased γδT17 cells in *Rpt* ^*f/f*^ *-Foxn1Cre* adult thymi could be selective expansion of these cells in the thymus after they were generated in the fetus and in neonate. However, thymic γδT cells or CD44^−^CD27^+^, CD44^+^CD27^+^, and CD44^+^CD27^−^ γδT subsets from *Rpt* ^*f/f*^ *-Foxn1Cre* mice incorporated BrdU at similar rates as their respective WT controls (Fig 10A–10C). Since γδT17 cells reside mainly in the CD44^+^CD27^−^ subset, these observations suggested that the relative increase of γδT17 cells was not likely caused by selective expansion of these cells in *Rpt* ^*f/f*^ *-Foxn1Cre* mice. Another potential but not mutually exclusive possibility was that mTORC1 deficiency in TECs caused selective retention of Vγ6^+^Vδ1^+^ γδT cell/γδT17 cells generated in the fetal thymus, leading to an increase of these cells in the adult *Rpt* ^*f/f*^ *-Foxn1Cre* thymus. To address this possibility, we generated chimerical mice by reconstituting lethally irradiated *Rpt* ^*f/f*^ and *Rpt* ^*f/f*^ *-Foxn1Cre* mice with bone marrow from CD45.1^+^CD45.2^+^ WT mice. Five to six weeks after transfer, *Rpt* ^*f/f*^ *-Foxn1Cre* recipient mice displayed a small thymus (Fig 10D), decreased total thymic total cellularity (Fig 10E) and comparable percentages, but decreased numbers of donor-derived CD45.1^+^ thymocyte subsets based on CD4 and CD8 staining (Fig 10F–10H). Donor-derived CD45.1^+^ γδT cell percentages were similar, but the numbers were decreased in *Rpt* ^*f/f*^ *-Foxn1Cre* recipients compared with *Rpt* ^*f/f*^ recipients (Fig 10I–10K). Importantly, γδT17 cell percentages were increased 4–8-fold in donor-derived γδT cells in the *Rpt* ^*f/f*^ *-Foxn1Cre* recipient thymus and spleen when compared with *Rpt* ^*f/f*^ recipients (Fig 10L). Thus, generation of fetal restricted γδT17 cells from WT hematopoietic stem cells from adult bone marrow was enhanced in adult thymi when mTORC1 was absent in TECs. Furthermore, percentages of Vγ5^+^ and Vγ6Vδ1^+^ cell in donor-derived γδT cells from *Rpt* ^*f/f*^ *-Foxn1Cre* recipient thymi appeared higher than those in donor-derived γδT cells from *Rpt* ^*f/f*^ recipient mice, while Vγ1.1^+^ and Vγ4^+^ γδT cell percentages were similar between these two groups (Fig 10M), suggesting that generation of fetal restricted Vγ5/Vγ6 γδT cells from WT hematopoietic stem cells from adult bone marrow might be increased in adult TEC specific mTORC1 deficient thymi.

### Decreased ETPs and Thymotropic Chemokines in *Rpt* ^*f/f*^ *-Foxn1Cre* Thymus

**Fig 11:**
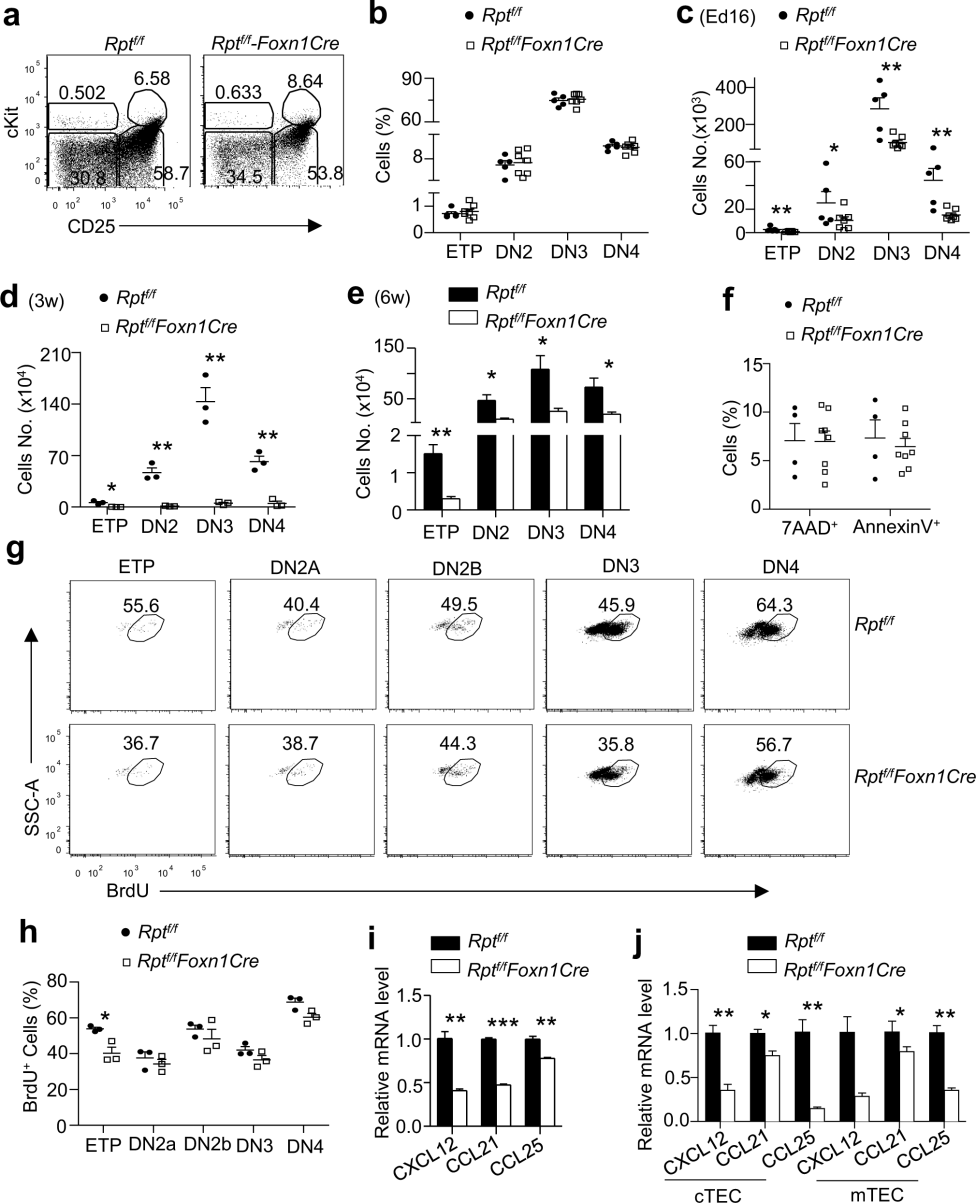
Decreased ETPs in mTORC1/Raptor-deficient thymi. **a.** Representative dot plots showing cKit and CD25 expression on live gated Lin^−^(CD4^−^CD8^−^CD3^−^TCRβ^−^B220^−^CD19^−^TCRγδ^−^NK1.1^−^CD11b^−^CD11c^−^Gr1^−^Ter119^−^) CD24^+^ thymocytes from E16 *Rpt* ^*f/f*^ and *Rpt* ^*f/f*^ *-Foxn1Cre* fetuses. The gating strategy is shown in S14A Fig. **b,c.** ETP and DN subset percentages (b) and numbers (c) in E16 fetuses. **d,e.** Numbers of ETP and DN subsets in 3-wk- (d) and 6-wk- (e) old *Rpt* ^*f/f*^ and *Rpt* ^*f/f*^ *-Foxn1Cre* mice. **f.** ETP death rate revealed by annexin V and 7-AAD staining. **g,h.** BrdU incorporation in ETP and DN subsets. Mice with 16-d pregnancy were i. p. injected with BrdU (3 mg in 200 μl PBS) and fetal thymi were harvested 6 h after injection for intracellular BrdU staining. The gating strategy is shown in S14B Fig. **g.** Representative dot plots of BrdU staining in gated subsets. **h.** BrdU percentages in the indicated DN subsets calculated from one litter of fetuses. **i,j.** Relative levels of thymotropic chemokines in E16 total thymi (i) and in sorted TECs from 10-d-old mice (j). Data shown represent or are calculated from at least three experiments (a–h) or two experiments (i,j). *, *p* < 0.05; **, *p* < 0.01; ***, *p* < 0.001 determined by two-tailed Student’s *t* test.

T cell development initiates after ETPs take residence in the thymus [10]. Severe impairment of multilineage T cell generation without obvious blockade at specific developmental stages prompted us to examine if ETPs in the thymus were altered in *Rpt* ^*f/f*^ *-Foxn1Cre* mice. The relative ratios of ETP (Lin^−^cKit^++^CD25^−^CD24^+^CD44^+^), DN2 (cKit^+^CD44^+^CD25^+^), DN3 (CD44^−^CD25^+^), and DN4 (CD44^−^CD25^−^) subsets within *Rpt* ^*f/f*^ *-Foxn1Cre* Lin^−^ thymocytes did not deviate greatly from E16 fetus, 3-wk- and 6-wk-old mice in the control group (Fig 11A and 11B). This suggests there was no obvious developmental blockade from ETP to DN4. However, ETPs and other DN subsets in fetal and postnatal *Rpt* ^*f/f*^ *-Foxn1Cre* thymi were decreased in numbers (Fig 11C–11E). *Rpt* ^*f/f*^ *-Foxn1Cre* ETPs showed no obvious defect in survival (Fig 11F). However, ETPs but not other DN subsets, displayed impaired BrdU incorporation (Fig 11G and 11H), suggesting decreased in vivo expansion of ETPs when mTORC1 was absent in TECs. Migration of ETPs to the thymus requires signals from CXCR4, CCR7, and CCR9, and their cognate ligands [37–40]. In E16 thymi (Fig 11I), as well as in sorted postnatal d10 TECs (Fig 11J), expression of these chemokines was considerably decreased in the absence of mTORC1. Together, these observations suggested that mTORC1 might, at least in part, enhance expression of multiple thymotropic chemokines in TECs for recruitment of ETPs to the thymus and promote ETP expansion for efficient T cell generation.

## Discussion

We demonstrated here that mTORC1 in TECs is pivotal for normal thymopoiesis and for establishing a thymic environment to foster proper T cell generation. Deficiency of mTORC1 in TECs resulted in severe thymic atrophy, decreased TEC numbers, abnormal thymic architecture, and decreased mTEC/cTEC ratios, leading to reduced ETPs in the thymus, impaired generation of virtually all T cell lineages, and defective temporal control of γδT17 differentiation and fetal restricted *TCRVγ/Vδ* recombination.

Using *Foxn1Cre*-mediated deletion, we have revealed that mTORC1 may control multiple aspects of TEC biology. First, mTORC1 is important for TEC expansion and its deficiency leads to decreased TEC numbers in both fetal and adult *Rpt* ^*f/f*^ *-Foxn1Cre* mice. Such function of mTORC1 in TECs is consistent with its role in cell cycle entry and synthesis of building blocks critical for cell growth and expansion [21]. Second, mTORC1 promotes late-stage TEC maturation indicated by decreased relative ratios of MHC-II^hi^CD40^hi^ TECs in *Rpt* ^*f/f*^ *-Foxn1Cre* mice. Although not examined in the current study, decreased mature TEC numbers likely affect thymic selection and T cell repertoire. Third, mTORC1 controls the balance between mTECs and cTECs and ensures establishing predominance of mTECs over cTECs. Finally, mTORC1 may augment the recruitment of ETPs to the thymus by increasing CXCL12, CCL21, and CCL25 expression in TECs and promote ETP expansion in the thymus through mechanism(s) yet to be defined.

Using *Rpt* ^*f/f*^ *-ERCre* mice, we have also shown that acute deletion of mTORC1 in adult mice quickly causes severe thymic atrophy, altered thymic architecture, and substantial reduction of DP thymocytes within one week. Our data are consistent with previous observations that rapamycin treatment induces thymic atrophy and DP thymocyte death in mice [41]. Since TEC numbers are not obviously reduced and thymocyte-specific deletion of *Raptor* does not affect thymus size and total cellularity [28], we propose that mature TEC function relies on mTORC1 activity to ensure DP thymocyte survival. Due to shortened life span of *Rpt* ^*f/f*^ *-ERCre* mice after tamoxifen injection, our results do not rule out a potential role of mTORC1 for mature c/mTEC homeostasis after prolonged deletion. It is important to point out that a recent study has found severe thymic atrophy in the same strain of mice 2–3 wk after tamoxifen injection and has attributed thymic atrophy to early T cell developmental blockade [28]. TECs and thymic architecture were not evaluated in that study. Our conclusion is not in conflict with that study, as hematopoiesis can be greatly impacted even after one week of tamoxifen treatment in these mice [42]. Nevertheless, to firmly establish the role of mTORC1 in mature TECs and how it promotes DP thymocytes survival, selective deletion of *Raptor* in mature TECs is required.

An important question that remains to be addressed is how mTORC1 controls TEC development and function. The transcription factor Foxn1 is essential for TEC development and thymopoiesis [7, 19] as well as for thymus maintenance [43, 44]. Aire is required for mTEC development and function [45]. Expression of these two molecules in TECs is not decreased in mTORC1 deficient mice (Fig 4E and S15 Fig). However, mTORC1 also controls nuclear translocation of multiple molecules [24], so our data do not rule out that mTORC1 may regulate the localization and function of these molecules. mTORC1 regulates the expression/activity of many other molecules [21]. The impairment of thymopoiesis and T cell development in mTORC1-deficient mice likely compounds the effects of multiple abnormalities.

Although the generation of virtually all T cell lineages is impaired in *Rpt* ^*f/f*^ *-Foxn1Cre* mice, individual T cell lineages appear to display differential sensitivities to mTORC1 deficiency in TECs. *i*NKT cells appear most stringently dependent on mTORC1 signaling in TECs, which is surprising, because their positive selection relies on engagement of the *i*Vα14TCR with self-lipid ligands presented by CD1d expressed on DP thymocytes in the cortex rather than TECs [32]. CD1d expression on and *Vα14-Jα18* recombination in DP thymocytes were not obviously affected in *Rpt* ^*f/f*^ *-Foxn1Cre* mice (S16 Fig). A recent study found that mTECs produce IL-15 to promote late stage *i*NKT cell development [33]. In *Rpt* ^*f/f*^ *-Foxn1Cre* mice, there was no obvious *i*NKT cell developmental blockade at a late stage, suggesting that mTORC1 in TECs may function through other mechanism(s) to promote early *i*NKT cell development. Similar to *i*NKT cells, nTregs are more sensitive than cαβT cells to mTORC1 deficiency. nTreg differentiation depends on self-peptide-MHC-II presented by or derived from mTECs [3, 4]. The disproportional decrease in the number of mTECs as well as reduction of mature mTECs in *Rpt* ^*f/f*^ *-Foxn1Cre* thymus may contribute to a more severe impairment of nTreg generation. Furthermore, a thymic environment such as local TGFβ and CD80/86-mediated costimulation modulates nTreg generation [46, 47]. Altered thymic environment in *Rpt* ^*f/f*^ *-Foxn1Cre* mice could also contribute to nTreg deficiency.

Immune cells undergo specific switches during development [48]. Among them are γδT17 cell differentiation and *TCRVγ5*, *Vγ6*, and *Vδ1* recombination, which predominantly or strictly occur in fetal thymus and are switched off in adult thymus [12–15]. Mechanisms that enforce such temporal controls or developmental switch are unknown. We demonstrated that TEC-specific deletion of mTORC1 results in a loss of fetal restriction on γδT17 differentiation and recombination of *Vγ5/6/Vδ1*, leading to uncontrolled γδT17-cell generation and *Vγ5/6Vδ1* recombination in adulthood. Our data suggest that mTORC1 controls TECs to enforce such temporal *Vγ5/6/Vδ1* recombination and γδT17 generation. Interestingly, a recent report has also found impaired temporal control of γδT17 differentiation and *Vγ/Vδ* recombination in adult β5t mutant mice [49]. Thus, although it has been previously suggested that fetal hematopoietic stem cells contain yet unknown properties that confer fetus specificity of *Vγ5/6/Vδ1* usages in an in vitro culture system [50], our data and those from Nitta et al. [49] suggest that thymic environment, particularly TECs, rather than fetal bone marrow hematopoietic stem cells (HSCs) ensures temporal control of γδT development. Interestingly, β5t mutation selectively impairs cTEC development to cause dysregulation of γδT17 cell generation in adult mice [49]. Because cTECs are relatively enriched in adult *Rpt* ^*f/f*^ *-Foxn1Cre* mice, it is possible that mTORC1 may play a crucial role in cTECs to restrain Vγ6^+^ γδT17 cell generation in adult thymi.

Important issues to be addressed in the future are how specific determinants in TECs dictate temporal control of γδT cell development and how mTORC1 signaling impact on these determinants. A potential possibility is that fetal and adult TECs are qualitatively different in a way that only fetal TECs confer an environment suitable for γδT17 differentiation and for opening *Vγ5/6Vδ1*chromatin for recombination. If this is true, mTORC1 may play an important role in the transition of TECs from fetal stage, which may be young and permissive for γδT17 differentiation and *TCRVγ5/6/Vδ1* recombination, to adult TECs, which are aged and impermissive for γδT17 differentiation and *TCRVγ5/6/Vδ1* recombination. γδT17 differentiation requires transcription factor RORγt and signals from Notch, TGFβ, and LTβR but not TCR [13, 51, 52] and is opposed by IL-15Rα signaling [53]. Whether mTORC1 acts on TECs to influence these signal mechanisms to confer temporal control of γδT17 differentiation is unknown at present. Nevertheless, our data provide genetic evidence that TECs nurture a thymic environment that limits postnatal γδT17 differentiation and *Vγ5/6Vδ1* recombination in an mTORC1-dependent manner.

Despite its importance, thymus undergoes involution or atrophy with advanced age or under certain pathological conditions. Thymic involution leads to a decrease in T cell production and shrinking of the T cell repertoire, which can result in impairment of adaptive immunity and propensity for autoimmunity [54, 55]. Altered TECs can either cause or prevent thymic involution/atrophy [43, 44, 56]. Given the roles of mTORC1 in TECs for thymopoiesis and thymus homeostasis, and the declination of mTORC1 signaling in TECs with age (Fig 1A), it is reasonable to speculate that gradual decreases of mTORC1 activity in TECs may contribute to thymic involution, and increases of mTORC1 activity might delay or prevent thymic involution. These hypotheses warrant further investigation. Additionally, rapamycin and its derivatives are utilized extensively in organ transplantation and cancer therapy. Their potential effects on thymic function should be taken into consideration.

## Materials and Methods

### Ethics Statement

Mouse experiments described were approved by the Institutional Animal Care and Use Committee of Duke University. Mice were euthanized with CO_2_ for experiments.

### Mice

*Rptor* ^*f/f*^ mice [57] were purchased from the Jackson laboratory and further backcrossed to C57Bl/6J background for at least four generations. *Foxn1Cre* mice [29] were kindly provided by Dr. Nancy Manley at the University of Georgia. *Rpt* ^*f/f*^-*Rosa26-ERCre* mice were previously reported [24, 58]. For ERCre mediated deletion, mice were i. p. injected with 200 μl 10 mg/ml tamoxifen on day 1, 2, and 5 and euthanized on day 8. Mice after overnight mating with virginal plug in the next morning were designated as gestation day 1. All animals were housed in specific pathogen-free conditions. Experiments described were approved by the Institutional Animal Care and Use Committee of Duke University.

### Preparation of TEC and Other Single Cell Suspension

TECs were prepared according to a published protocol with modifications [59]. In brief, thymi were cut into 2 mm pieces and directly digested in 2 ml digestion buffer (250 μl 10mg/ml collagenase type IV (Worthington), 40 μl 50mg/ml DNase I (Worthington) and 1.71ml FBS-free RPMI-1640) at 37°C with constant orbital shaking at 150–200 rpm for 15 min. After gentle vortex, remnants were allowed to settle down; the supernatants were collected and kept on ice; settled remnants were digested similarly two more times. After the last digestion, cells were combined and filtered through 70 μm nylon mesh. After centrifuged at 472 g for 5 min, pellets were resuspended in 10 ml RPMI-containing 10% FBS (RPMI-10), spun again, and resuspended in either cold FACS buffer (5 Mm EDTA, 2%FBS in PBS) or RPMI-10. Newborn and fetal thymi were treated similarly except that 500 μl of digestion buffer was used. TECs used for sorting were enriched by EasySep APC positive selection Kit (Stemcell Technologies) after staining with an APC-conjugated anti-EpCAM antibody.

Total lung cells were isolated according to a published protocol with modifications[60]. Briefly, lung was cut into approximately 1–2 mm pieces and then digested in 2 ml digestion buffer (500 μl 10 mg/ml collagenase type IV, 10 μl 50 mg/ml DNase I and 1.5ml 5% FBS IMDM) at 37°C for 1 h with shaking every 10 min. Cells were washed with, and resuspended in, IMDM containing 5%FBS. Liver mononuclear cells were isolated using gradient centrifugation as previously described [61].

### Antibodies and Flow Cytometry

Fluorochrome-conjugated anti-CD45.2 (clone 104), CD45(clone 30-F11), CD45.1(clone A20), EpCAM/CD326 (clone G8.8), Ly51 (clone 6C3), MHCⅡ-I-A/I-E (clone M5/114.15.2), CD40 (clone 3/23), CD4 (clone GK1.5), CD8 (clone 53–6.7), TCR-β (clone H57-597), TCRγδ (clone GL3, NK1.1 (clone PK136), CD44 (clone IM7), CD24 (clone M1/69), CD25 (clone PC61.5), CD27 (clone LG.3A10), c-Kit/CD117 (clone 2B8), CD62L (clone MEL-14),Gr1 (clone RB6-8C5), CD11b (clone M170), CD11c (clone N418), F4/80 (clone BM8), CD1d (clone 1B1), CD1d tetramer (NIH tetramer facility), B220 (clone RA3-6B2), CD19 (clone 6D5), TER119/Erythroid Cells (clone TER-119), CD3e (clone 145-2C11), Annexin-V, Streptavidin,CCR6 (clone 29-2L17), IFN-γ (clone XMG1.2), IL-17A (clone TC11-18 H10.1), Foxp3 (clone FJK-61s, eBioscience), Anti-TCR-Vγ1.1 (clone 2.11, BioXcell), Vγ4 (clone UC3-10A6, BioXcell), Vγ5 (clone 536), Vγ7 (clone F2.67), and Vδ6.3 (clone 17C, kindly provided by Dr. Pablo Pereira, Institut Pasteur, France) were purchased from Biolegend unless indicated otherwise. Ulex Europaeus Agglutinin I (UEA-1, clone B-1065) was purchased from vector laboratories. Phospho-S6 (Ser235/236, d57.2.2E) antibody was from Cell Signal Technology. FITC-conjugated TCR-Vβ usage kit, including anti-TCRβ2 (clone B20.6), β3 (clone KJ25), β4 (clone KT4), β5.1/5.2 (clone MR9-4), β6 (clone RR4-7), β7 (clone TR310), β8.1/8.2 (clone MR5-2), β8.3 (clone IB3.3), β9 (clone MR10-2), β10^b^ (clone B21.5), β11 (clone RR3-15), β12 (clone MR11-1), β13 (clone MR12-3), β14 (clone 14–2), and β17^a^ (clone KJ23) were purchased from BD Pharmingen. The 17D1 (anti-Vγ5Vδ1) monoclonal antibody was prepared from cultured hybridoma supernatant after ammonium sulfate precipitation and affinity purification with a goat anti-rat IgM (Jackson ImmunoResearch Laboratories) conjugated Sepharose-4B beads (Amersham Pharmacia Biotech AB) and was conjugated with biotin (ProteoChem) according to a manufacturer’s protocol. The 17D1 antibody detects Vγ6Vδ1 when pretreated with the anti-TCRγδ (GL3) antibody [62]. Cells were stained for cell surface molecules using 2% FBS-PBS. Cell death was identified by using the Violet Live/Dead cell kit (Invitrogen) or annexin-V and 7-AAD. Intracellular staining for Foxp3 was performed using the eBioscience Foxp3 Staining Buffer Set. Phospho-S6 staining was performed using the BD Biosciences Cytofix/Cytoperm and Perm/Wash solutions. Stained samples were acquired on a FACS Canto-II (BD Biosciences) flow cytometer. Data was analyzed with FlowJo software (Tree Star). All FCS files associated with data presented in this study have been deposited in the zenodo website (http://zenodo.org/record/34843 or DOI URL: http://dx.doi.org/10.5281/zenodo.34843).

### Stimulation and Intracellular Cytokine Detection

Thymocytes and single cell suspensions from other organs were stimulated with phorbol 12-myrustate 13-acetatae (PMA, 50 ng/ml) plus ionomycin (500 ng/ml) in the presence of GolgiPlug (1 ng/ml) for 4 h. After cell surface staining, intracellular staining for IL-17A and IFN-γ was performed by using the BD Biosciences Cytofix/Cytoperm and Perm/Wash solutions.

### BrdU Incorporation

For thymocytes, mice were i. p. injected with 5-bromo-2-deoxyuridine (BrdU, Sigma; 1 mg/mouse or 50 mg/kg in 100–200 μl PBS) and were stained 4 h after injection to assess BrdU incorporation. For embryonic ETPs, mice pregnant for 16 d were i. p. injected with 3 mg BrdU, and fetal thymi were collected 6 h after injection to assess BrdU incorporation. For TECs, mice were i. p. injected with 1 mg BrdU 3 times every 24 h, and thymi were collected 14 h after the last injection for TEC preparation. After cell surface staining, cells were intracellularly stained for BrdU using a BrdU Flow Kit (BD Biosciences) according to the manufacturer’s protocol.

### Histology and Immunofluorescence Microscopy

Thymus for H&E staining were fixed in 10% formalin solution for 1 d, and then changed into 70% ethanol. Paraffin-thin sections were stained with H&E according to standard protocols. Thymus for immunofluorescent staining were embedded in OCT (Leica Biosystems Richmond Inc, Richmond) and frozen immediately in −80°C. Frozen thin sections (5 μm) were fixed in a 1:1 mixture of acetone and methanol at −20°C for 8 min. After blocking with PBS containing 3% BSA with 0.1% Tritonx-100 for 30–45 min at room temperature (RT), samples were stained using primary rat-anti-mouse-keratin 8 (Troma-1, DSHB, University of Iowa, 1:50 dilution) and rabbit-anti-mouse-keratin 5 (PRB-160P, Covance; 1:200 dilution), followed by secondary Rhodamine-conjugated-donkey anti-rabbit IgG (1:400 dilution) and FITC-conjugated-goat anti-rat IgG (1:400 dilution, Santa Cruz Biotechnology). Samples were mounted with Vector mounting solution containing DAPI (Vector) and allowed to dry overnight at RT or 4°C in the dark before imaging. Images were acquired using a Zeiss ApoTome Microscope and analyzed using PhotoshopCS4 software.

### Bone Marrow Reconstitution and Generation of Irradiation Chimeric Mice

CD45.1^+^CD45.2^+^ WT bone marrow cells were depleted off T cells using a PE-conjugated anti-CD3 antibody, anti-PE-antibody conjugated magnetic beads, and LD columns (Miltenyi Biotec) according to the manufacturer’s protocol. CD45.2^+^ *Rpt* ^*f/f*^ and *Rpt* ^*f/f*^ *-Foxn1Cre* mice were lethally irradiated (1000 rad) and were intravenously injected with 1.0 x 10^7^ T cell depleted bone marrow cells 4 h after irradiation. Recipient mice were analyzed 5–6 wk later.

### TEC Glucose Uptake Assay

Single cell suspension made for TEC preparations from 3-wk-old *Rpt* ^*f/f*^ *-Foxn1Cre* and WT littermates were plated at 1 x 10^7^ cells/well in 96-well U-bottom plates and treated with or without fluorescent 100 μM 2-(N-(7-Nitrobenz-2-oxa-1, 3-diazol-4-yl)Amino)-2-Deoxyglucose (2-NBDG; Life Technologies) in PBS and incubated at 37°C with 5% CO_2_ for 30 min. The 2-NBDG uptake reaction was stopped by removing culture medium and washed with pre-clod PBS two times. Cells were stained for surface molecules before analysis with flow cytometry.

### Quantitative Real-Time and Semiquantitative PCR

Total RNAs were extracted from E16 thymi or sorted 10 d-old mTECs (Epcam^+^CD45^−^UEA-1^+^Ly51^−^) and cTECs (Epcam^+^CD45^−^UEA-1^+^Ly51^−^) using the Trizol reagent (Sigma). The first strand cDNAs were reversely transcribed using an iScript cDNA Synthesis Kit (Bio-Rad). Quantitative real-time PCR (qRT-PCR) was performed using a Mastercycler Realplex (Eppendorf) and the SensiMix SYBR No-ROX Kit (Bioline). Data were analyzed using the 2^-Ct^ method after normalization to *β*-*actin* expression and shown as relative expression levels. For semi-quantitative PCR, cDNA template in 1:4 serial dilutions from each sample was used. For Vα14 to Jα2, Jα18, and Jα56 recombination, genomic DNA isolated from sorted CD4^+^CD8^+^ DP thymocytes from *Rpt* ^*f/f*^ and *Rpt* ^*f/f*^ *-Foxn1Cre* mice was used as the template for semi-quantitative PCR according to a previously published protocol except that *TSC1* was used as loading control [61]. Primer pairs used for the amplification are summarized in Table 1.

**Table 1:** Primer pairs.

Gene	Forward Primer 5’-3’	Reverse Primer5’-3’
*CXCL12*	GTCCTCTTGCTGTCCAGCTC	GGTAGCTCAGGCTGACTGGT
*CCL21*	TCCGAGGCTATAGGAAGCAA	CTTCCTCAGGGTTTGCACAT
*CCL25*	TCACCAGCAGGAAGTGAGTG	GATTCTCATCGCCCTCTTCA
*IL-7*	ATTATGGGTGGTGAGAGCCG	GTTCCTGTCATTTTGTCCAATTCA
*Foxn1*	TGACGGAGCACTTCCCTTAC	GACAGGTTATGGCGAACAGAA
*Vα14-Jα2*	ACACTGCCACCTACATCTGT	GGTTGCAAATGGTGCCACTT-3
*Vα14-Jα18*	ACACTGCCACCTACATCTGT	GTAGAAAGAAACCTACTCACCA
*Vα14-Jα56*	ACACTGCCACCTACATCTGT	TGTCATCAAAACGTACCTGGT
*TSC1*	GTCACGACCGTAGGAGAAGC	GAATCAACCCCACAGAGCAT
*TCRVγ1-Jγ4*	TTGGTACCGGCAAAAAGCAAAAA	GGCACATCATGGGTCAAGAT
*TCRVγ2-Jγ2*	CTGTTGATTTGTTTTTTGCCGG	TCTGCAAATACCTTGTGAAAGCCCGAGCTAT
*TCRVγ6-Cγ1*	GGACATGGCAGAGTGATTTG	GGAAGGAAAATAGTGGGCTTG
*TCRVγ5-Cγ1*	ACTCCCGCTTGGAAATTGAT	TGTCTGCATCAAGCCTTTTG
*TCRVγ4-Cγ1*	TGCAACCCCTACCCATATTT	TGTGGTGGATTCCAGATTCA
*TCRVδ1-Cδ*	ATTCAGAAGGCAACAATGAAAG	ATGATGAAAACAGATGGTTTGG
*β-actin*	TGTCCACCTTCCAGCAGATGT	AGCTCAGTAACAGTCCGCCTAGA

### Statistical Analysis

Data were presented as mean ± SEM and analyzed for statistical differences using the Prism 5/GraphPad software. Comparisons were made using two-tailed Student’s *t* test. *p*-Values less than 0.05 were considered significant.

## Supporting Information

S1 Data Raw data for analyses shown in Figures and Supplemental Figures of the manuscript. (XLSX)

S1 Fig Gating strategies for FACS plots in Fig 1. **A.** Gating strategies to get to TECs for Fig 1A. **B.** Gating strategies to get to live thymocytes for Fig 1D. **C.** Gating strategies to get to thymocytes for Fig 1F. **D.** Gating strategies to get to TECs for Fig 1I and 1K.
(PDF)

S2 Fig Gating strategies for FACS plots in Fig 3. Gating strategies to get to TECs for Fig 3A, 3F and 3G.
(PDF)

S3 Fig Effects of Cre expression in TECs on thymopoiesis and T cell development. *Foxn1Cre* ^*+*^ and *Foxn1Cre* ^*−*^ 3-wk-old litter-mates were examined. **A.** Thymus size. **B.** Total thymic cellularity. Each circle or square represents one *Foxn1Cre* and WT, respectively. Bars represent mean ± SEM. **C.** Representative dot plots of thymocytes. **D.** Percentages and numbers of thymocyte subsets. **E.** Representative dot plots of TECs. **F.** Percentages and numbers EpCAM^+^CD45^−^ TECs in thymus. **G.** Percentages and numbers of mTECs and cTECs. **H.** MHCII and CD40 staining of gated mTECs. Data shown represent three experiments (WT, *n* = 5; KO, *n* = 5).
(PDF)

S4 Fig Gating strategies for FACS plots in Fig 4A, 4C and 4F. (PDF)

S5 Fig Gating strategies for FACS plots in Fig 5. **A.** Gating strategy for Fig 5A. **B.** Gating strategy for Fig 5H.
(PDF)

S6 Fig Assessment of TCRVβ usages. Splenocytes from *Rpt* ^*f/f*^ and *Rpt* ^*f/f*^ *-Foxn1Cre* mice were stained with CD4, CD8, and individual TCRVβ chains using a TCRβ staining kit (BD Biosciences). Bar graphs represent mean ± SEM of individual TCRβ chain percentages in gated CD4 or CD8 T cells.
(PDF)

S7 Fig Gating strategies for FACS plots in Fig 6. **A.** Gating strategy for Fig 6A and 6D. **B.** Gating strategy for Fig 6H. **C.** Gating strategy for Fig 6I and 6M.
(PDF)

S8 Fig Gating strategies for FACS plots in Fig 7. **A.** Gating strategy for Fig 7A. **B.** Gating strategy for Fig 7E.
(PDF)

S9 Fig Gating strategies for FACS plots in Fig 8. **A.** Gating strategy for Fig 8A. **B.** Gating strategy for Fig 8E.
(PDF)

S10 Fig Effects of Cre expression in TECs on γδT cell development. *Foxn1Cre* ^*+*^ and *Foxn1Cre* ^*−*^ 3-wk-old litter-mates were examined. **A.** γδT percentages and numbers in the thymus. **B.** Representative dot plots of IL-17A and IFNγ staining in thymic γδT cells. Thymocytes were stimulated with PMA plus ionomycin in the presence of brefeldin A (BFA) for 4 h followed by cell surface and intracellular staining. Dot plots show IL-17A and IFNγ expression in gated TCRγδ^+^TCRβ^−^ cells. **C.** γδT1 and γδT17 percentages and numbers in the thymus. **D.** γδT1 and γδT17 numbers in the thymus. Data shown represent three experiments (WT, *n* = 5; KO, *n* = 5).
(PDF)

S11 Fig Gating strategies for FACS plots in Fig 9. **A.** Sorting strategy for γδT cells used in Fig 9A and 9B. **B.** Gating strategy for Fig 9D. **C.** Gating strategy for Fig 9F.
(PDF)

S12 Fig Representative dot plots showing TCRγδ and indicated Vγ staining in gated TCRγδ^+^TCRβ^−^ thymocytes from newborn *Rpt* ^*f/f*^ and *Rpt* ^*f/f*^ *-Foxn1Cre* mice. Data shown represent three experiments.
(PDF)

S13 Fig Gating strategies for FACS plots in Fig 10. **A.** Gating strategy for Fig 10A. **B.** Gating strategy for Fig 10F. **C.** Gating strategy for Fig 10I. **D.** Gating strategy for Fig 10M.
(PDF)

S14 Fig Gating strategies for FACS plots in Fig 11. **A.** Gating strategy for Fig 11A. **B.** Gating strategy for Fig 11G.
(PDF)

S15 Fig Foxn1 expression in TECs. Relative Foxn1 mRNA levels in sorted TECs from 10-d-old mice were determined by real-time qPCR.
(PDF)

S16 Fig iVα14-Jα18 recombination and CD1d expression in DP thymocytes. **A.** Genomic DNA isolated from sorted CD4^+^CD8^+^ DP thymocytes from *Rpt* ^*f/f*^ and *Rpt* ^*f/f*^ *-Foxn1Cre* mice were utilized for detection of Vα14 to Jα2, Jα18, and Jα56 recombination using semi-quantitative PCR. *TSC1* was used as loading control. **B.** Overlaid histograms show CD1d expression on DP thymocytes. Data shown represent three experiments.
(PDF)

## Abbreviations

BFA: brefeldin A
cαβT: conventional TCRα/β T
cTEC: cortical thymic epithelial cell
DN: double negative
DP: double positive
ETP: early T cell progenitor
FACS: fluorescence-activated cell sorting
FITC: fluorescein isothiocyanate
H&E: hematoxylin and eosin
HSC: hematopoietic stem cell
*i*NKT: invariant Vα14-Jα18 TCR-expressing NKT
i. p.: intraperitoneally
KO: knockout
MHC: major histocompatibility complex
mTEC: medullary thymic epithelial cell
mTOR: mammalian/mechanistic target of rapamycin
mTORC1: mammalian/mechanistic target of rapamycin complex 1
qRT-PCR: quantitative real-time PCR
RT: room temperature
PMA: phorbol myristate acetate
SEM: standard error of the mean
SP: single positive
TEC: thymic epithelial cell
TRA: tissue-specific antigen
Treg: regulatory T cell
WT: wild-type
