# Supplementary figures and images for: mTORC1 in Thymic Epithelial Cells Is Critical for Thymopoiesis, T-Cell Generation, and Temporal Control of γδT17 Development and TCRγ/δ Recombination
Source: PLoS Biol. 2016 Feb 18;14(2):e1002370. doi: 10.1371/journal.pbio.1002370 (PMC4758703; doi:10.1371/journal.pbio.1002370)

A

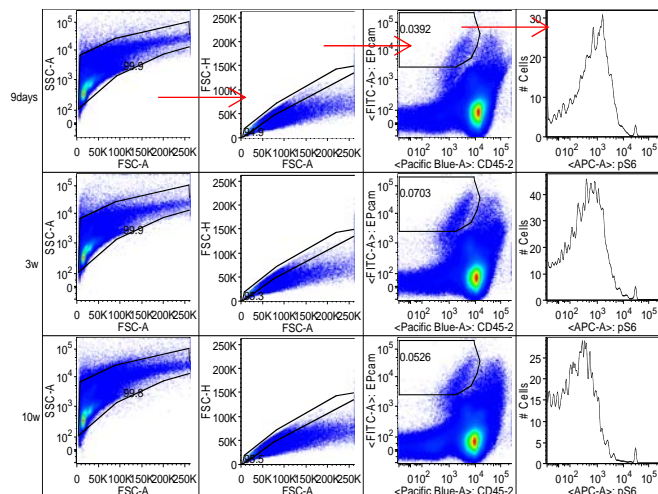

C

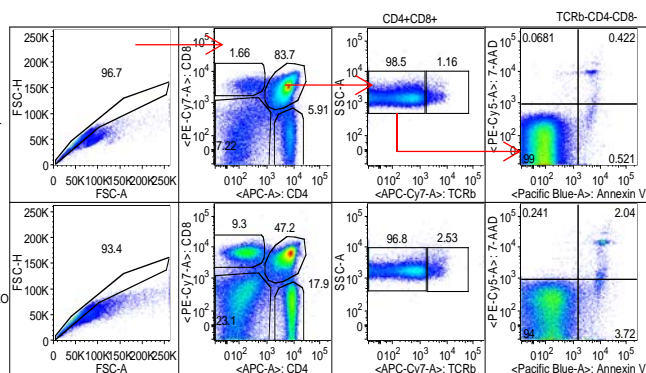

B

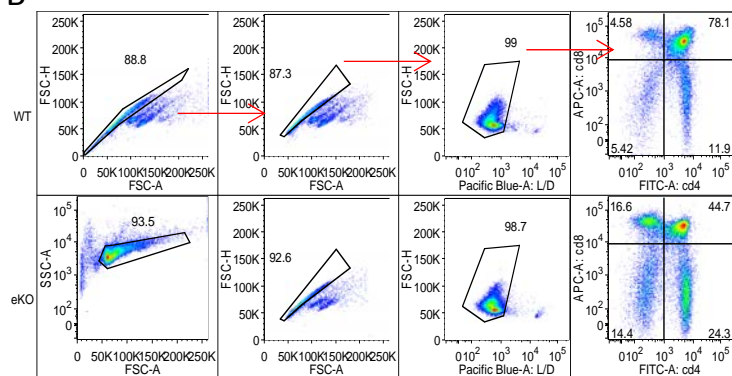

D

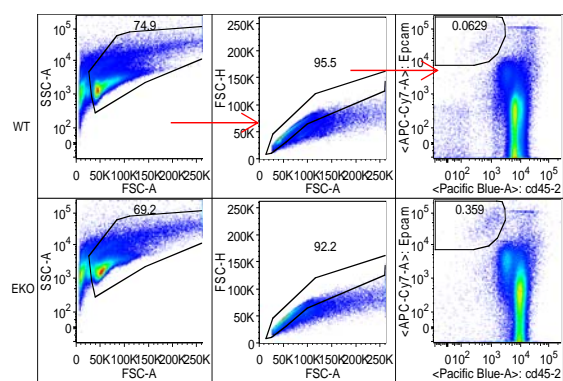

Supplement: S1 Fig — A. Gating strategies to get to TECs for Fig 1A. B. Gating strategies to get to live thymocytes for Fig 1D. C. Gating strategies to get to thymocytes for Fig 1F. D. Gating strategies to get to TECs for Fig 1I and 1K. (PDF) [file pbio.1002370.s002.pdf]

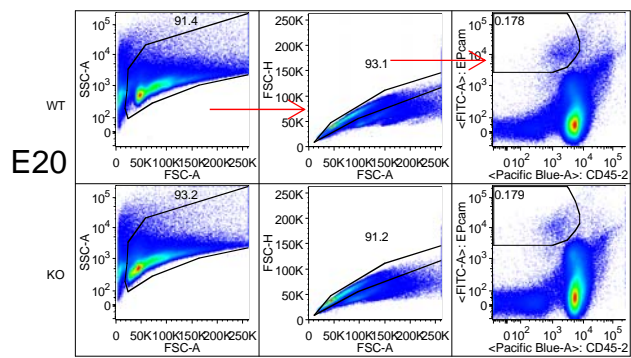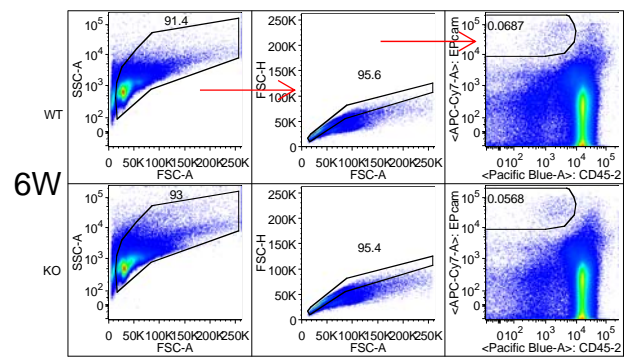

Supplement: S2 Fig — Gating strategies to get to TECs for Fig 3A, 3F and 3G. (PDF) [file pbio.1002370.s003.pdf]

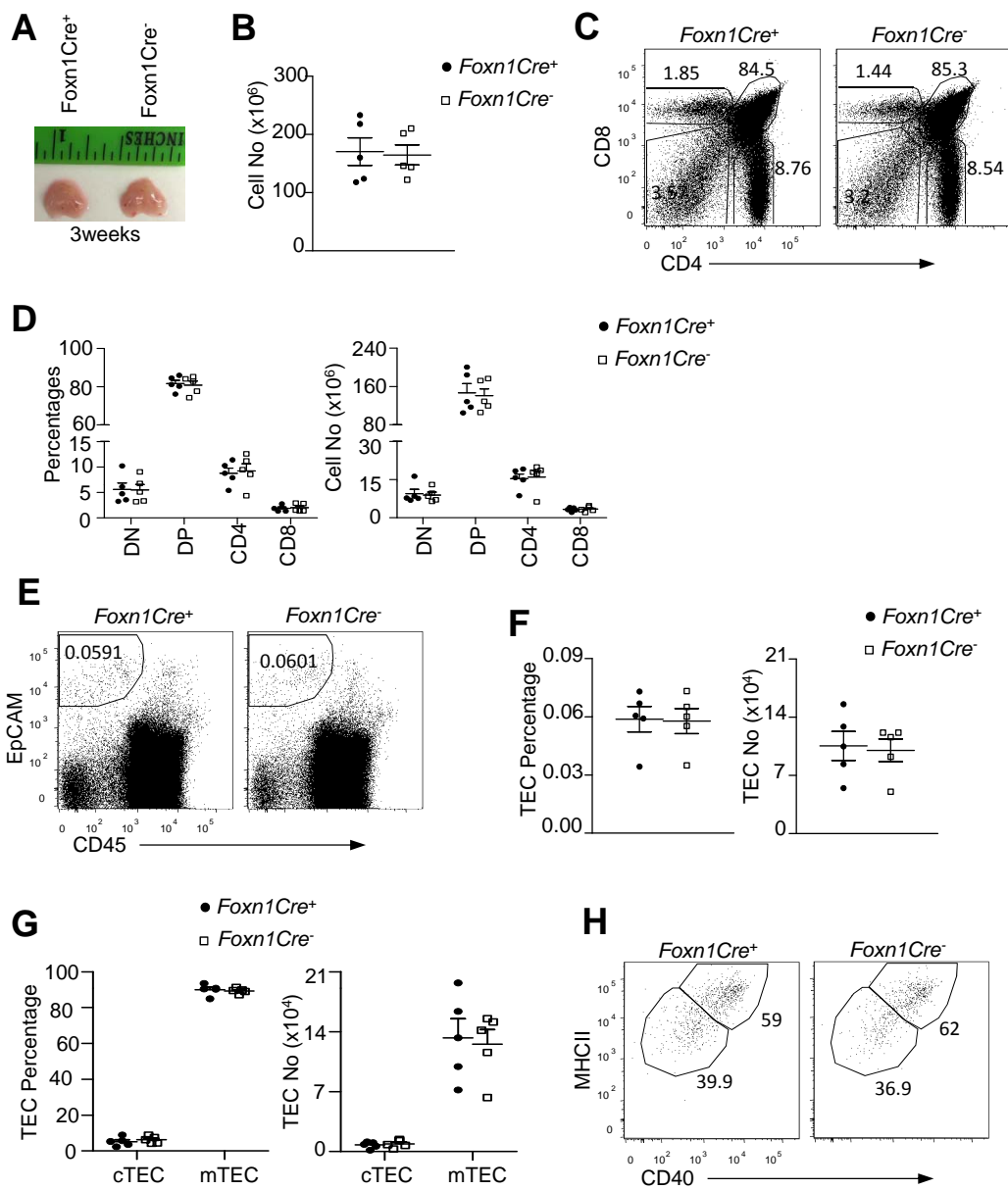

Supplement: S3 Fig — Foxn1Cre + and Foxn1Cre − 3-wk-old litter-mates were examined. A. Thymus size. B. Total thymic cellularity. Each circle or square represents one Foxn1Cre and WT, respectively. Bars represent mean ± SEM. C. Representative dot plots of thymocytes. D. Percentages and numbers of thymocyte subsets. E. Representative dot plots of TECs. F. Percentages and numbers EpCAM+CD45− TECs in thymus. G. Percentages and numbers of mTECs and cTECs. H. MHCII and CD40 staining of gated mTECs. Data shown represent three experiments (WT, n = 5; KO, n = 5). (PDF) [file pbio.1002370.s004.pdf]

3w

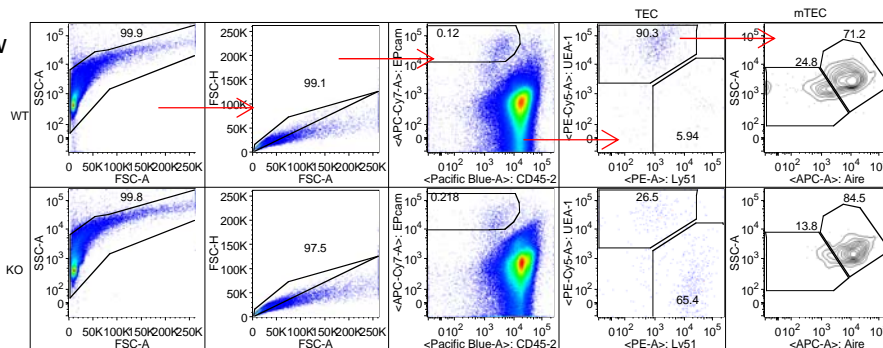

6-8w

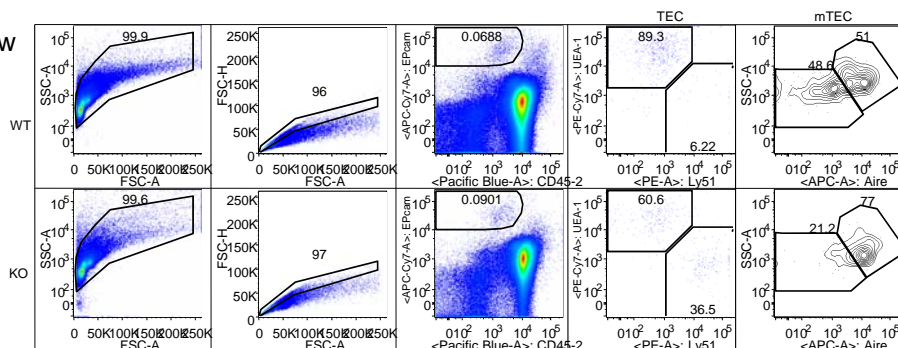

Supplement: S4 Fig — (PDF) [file pbio.1002370.s005.pdf]

## A thymus

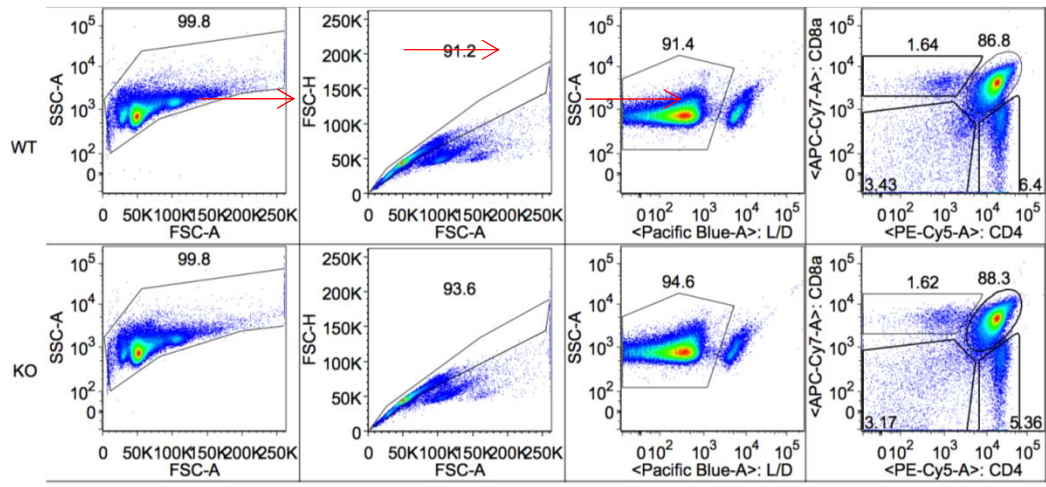

## B spleen

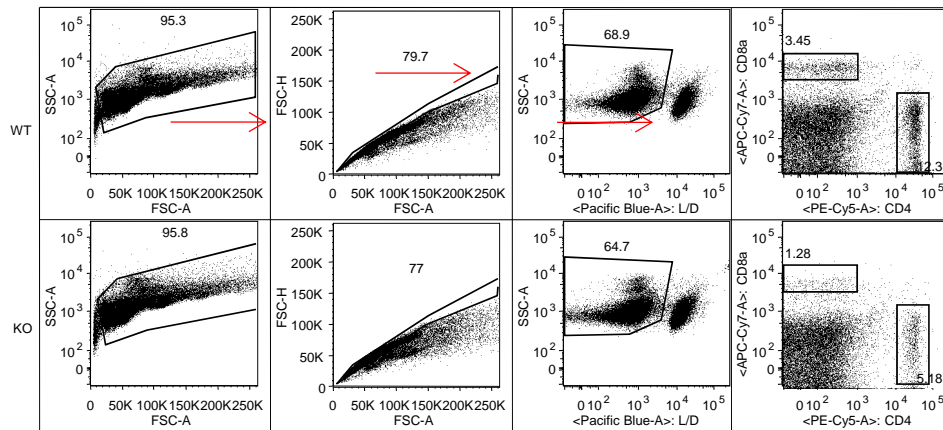

Supplement: S5 Fig — A. Gating strategy for Fig 5A. B. Gating strategy for Fig 5H. (PDF) [file pbio.1002370.s006.pdf]

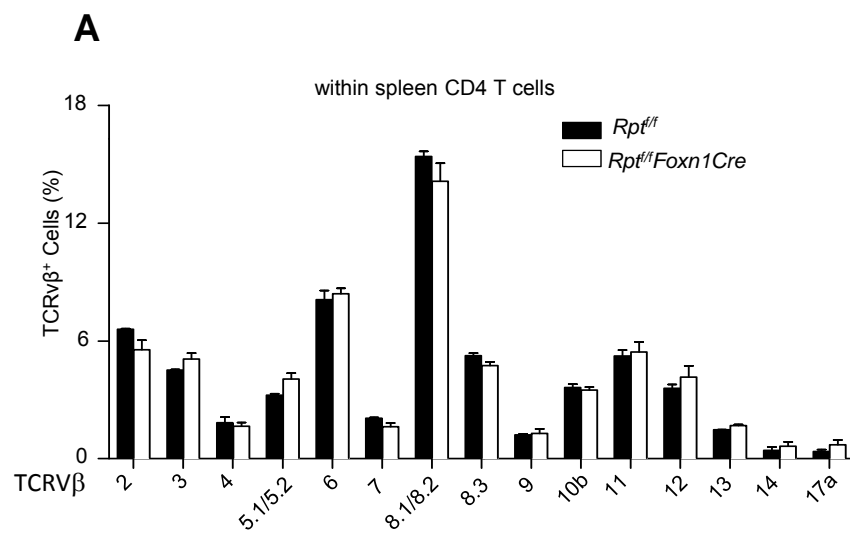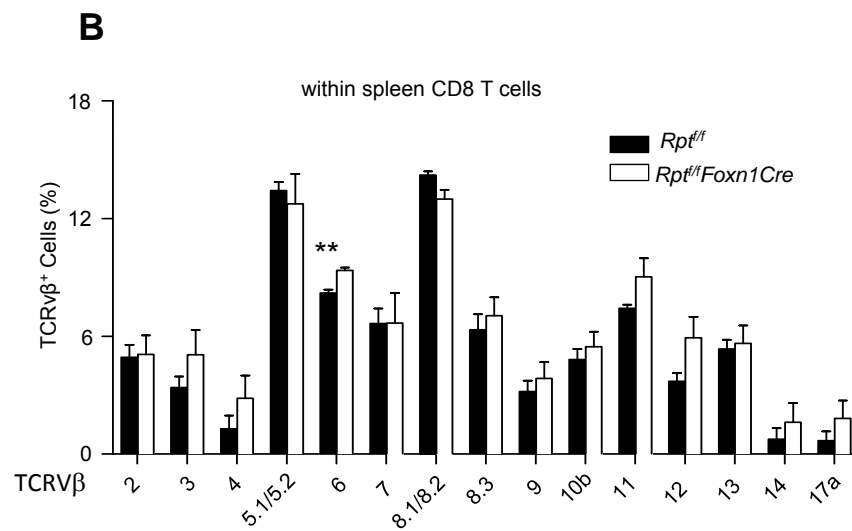

Supplement: S6 Fig — Splenocytes from Rpt f/f and Rpt f/f -Foxn1Cre mice were stained with CD4, CD8, and individual TCRVβ chains using a TCRβ staining kit (BD Biosciences). Bar graphs represent mean ± SEM of individual TCRβ chain percentages in gated CD4 or CD8 T cells. (PDF) [file pbio.1002370.s007.pdf]

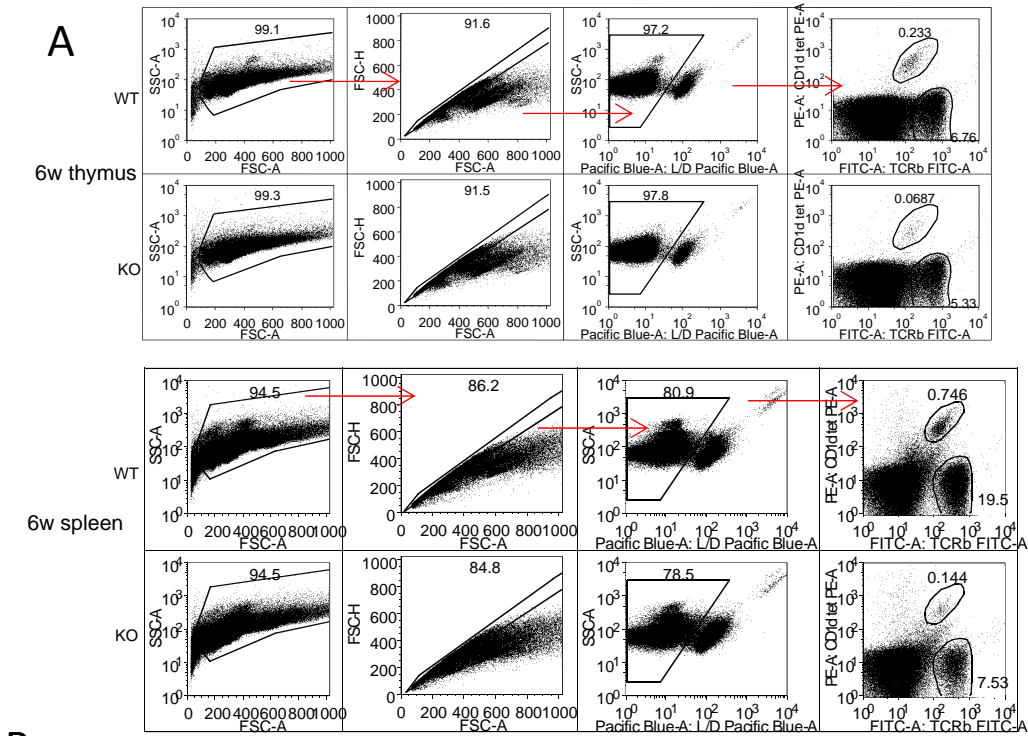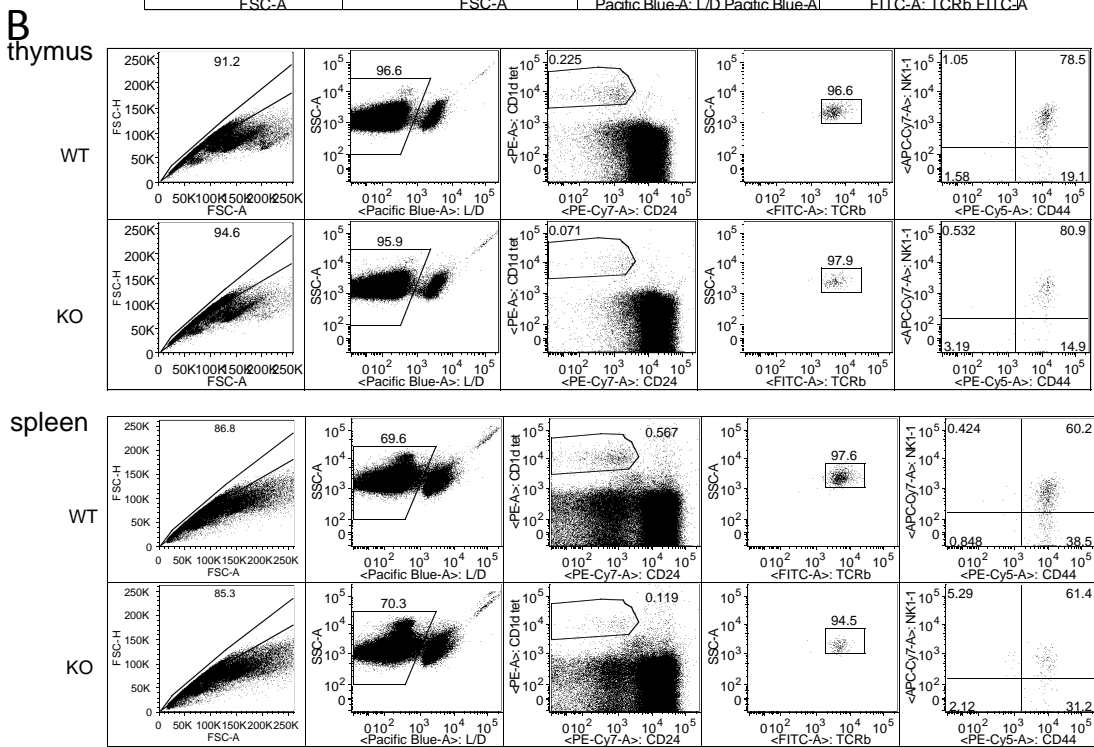

Supplement: S8 Fig — A. Gating strategy for Fig 7A. B. Gating strategy for Fig 7E. (PDF) [file pbio.1002370.s009.pdf]

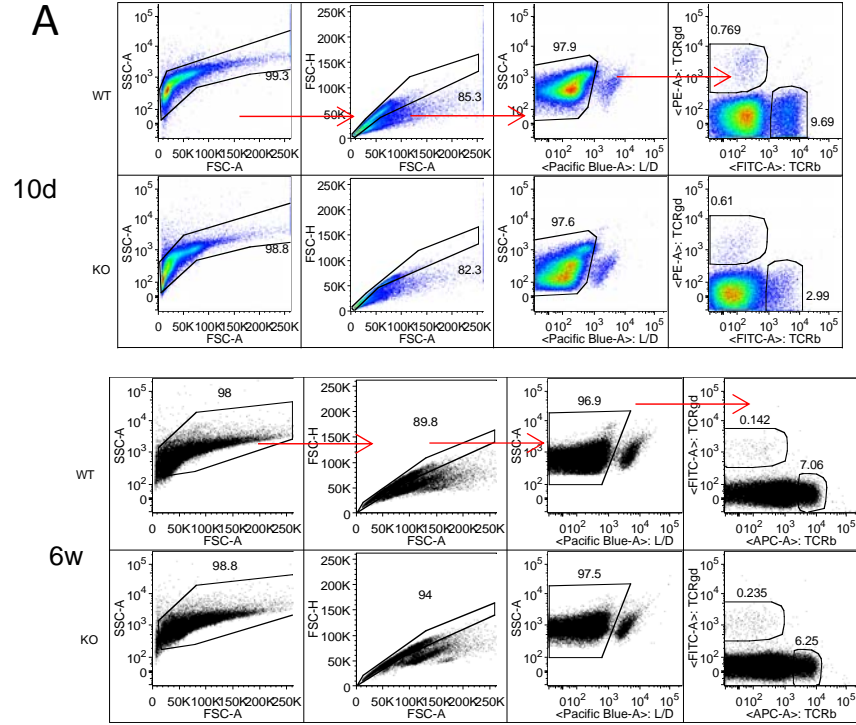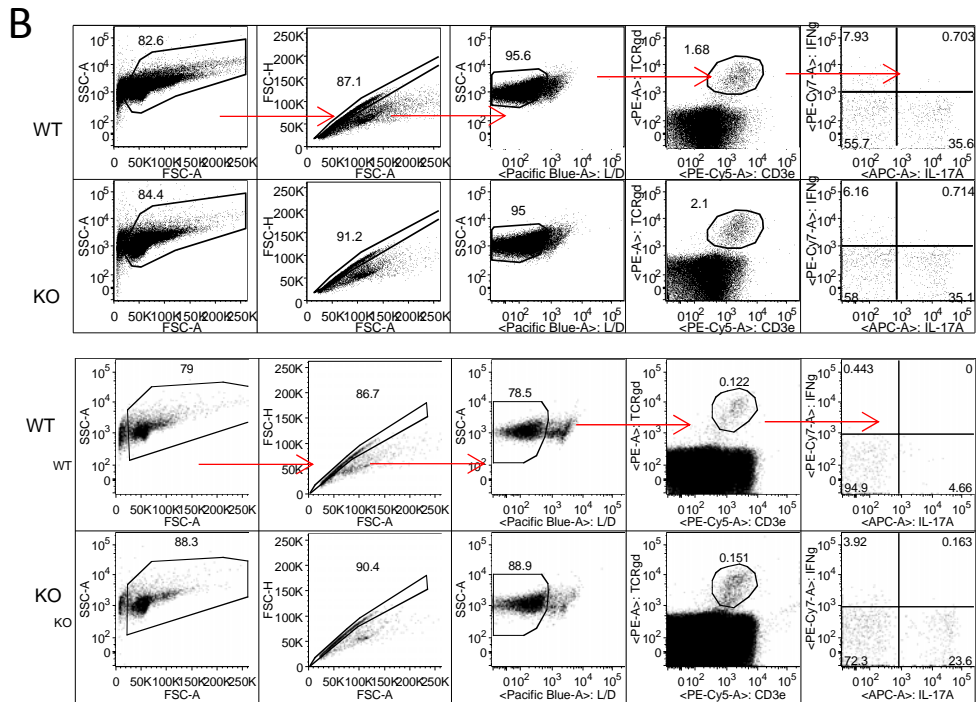

Supplement: S9 Fig — A. Gating strategy for Fig 8A. B. Gating strategy for Fig 8E. (PDF) [file pbio.1002370.s010.pdf]

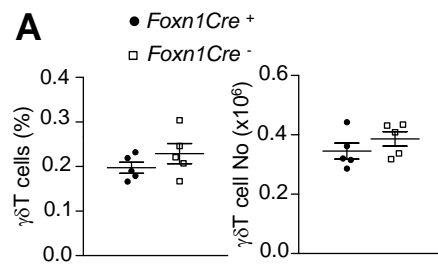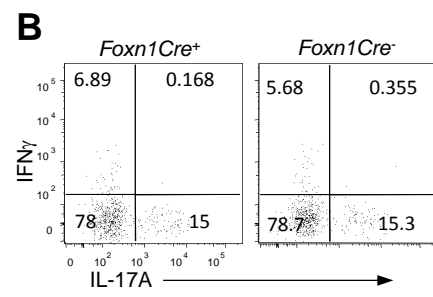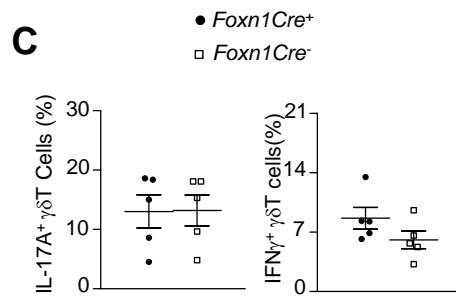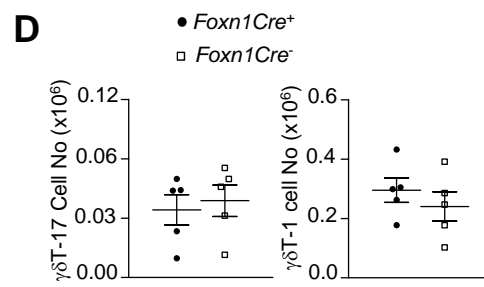

Supplement: S10 Fig — Foxn1Cre + and Foxn1Cre − 3-wk-old litter-mates were examined. A. γδT percentages and numbers in the thymus. B. Representative dot plots of IL-17A and IFNγ staining in thymic γδT cells. Thymocytes were stimulated with PMA plus ionomycin in the presence of brefeldin A (BFA) for 4 h followed by cell surface and intracellular staining. Dot plots show IL-17A and IFNγ expression in gated TCRγδ+TCRβ− cells. C. γδT1 and γδT17 percentages and numbers in the thymus. D. γδT1 and γδT17 numbers in the thymus. Data shown represent three experiments (WT, n = 5; KO, n = 5). (PDF) [file pbio.1002370.s011.pdf]

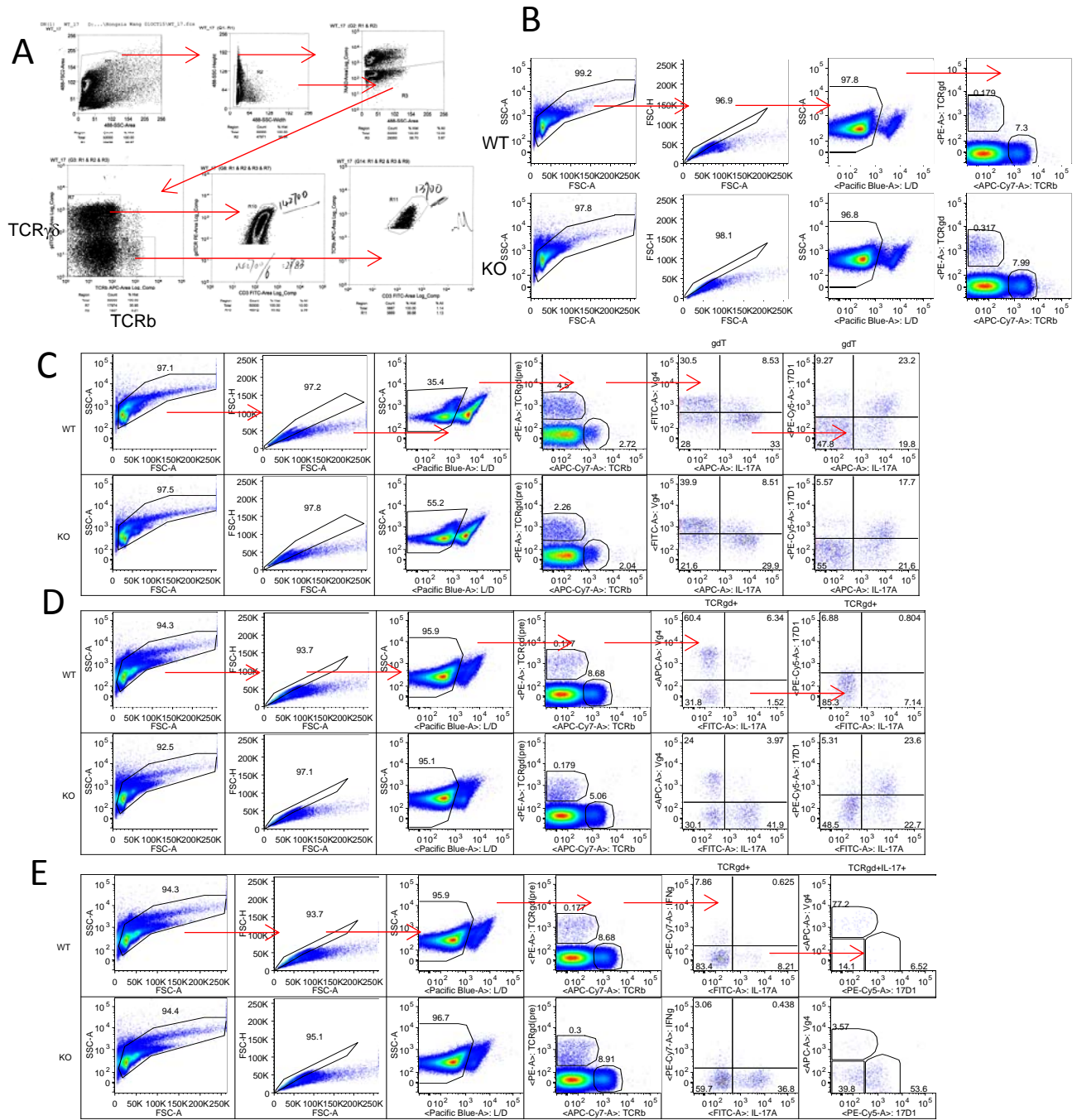

Supplement: S11 Fig — A. Sorting strategy for γδT cells used in Fig 9A and 9B. B. Gating strategy for Fig 9D. C. Gating strategy for Fig 9F. (PDF) [file pbio.1002370.s012.pdf]

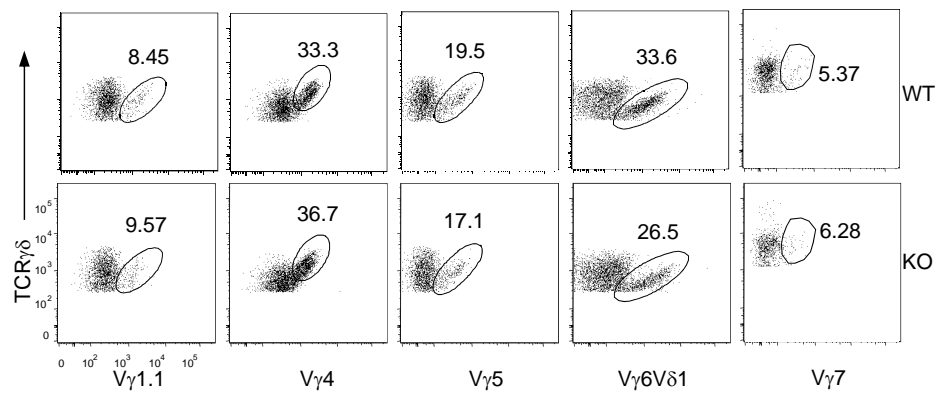

Supplement: S12 Fig — Data shown represent three experiments. (PDF) [file pbio.1002370.s013.pdf]

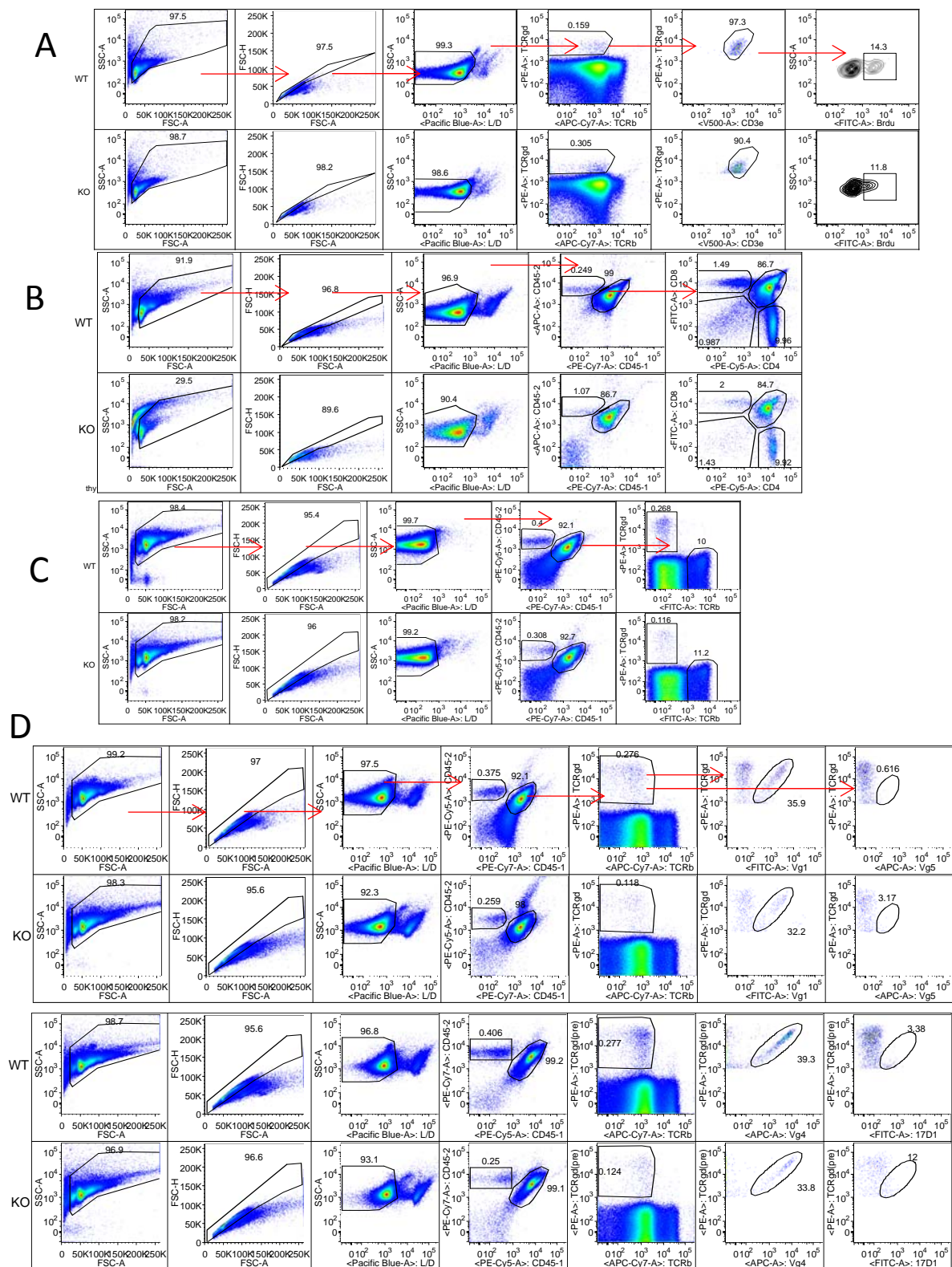

Supplement: S13 Fig — A. Gating strategy for Fig 10A. B. Gating strategy for Fig 10F. C. Gating strategy for Fig 10I. D. Gating strategy for Fig 10M. (PDF) [file pbio.1002370.s014.pdf]

A

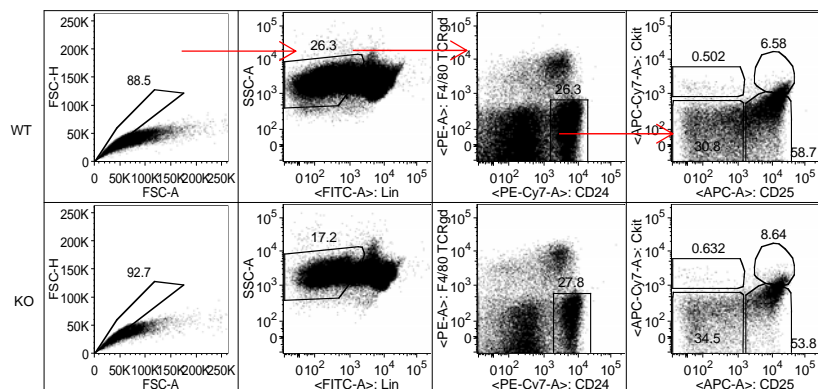

B

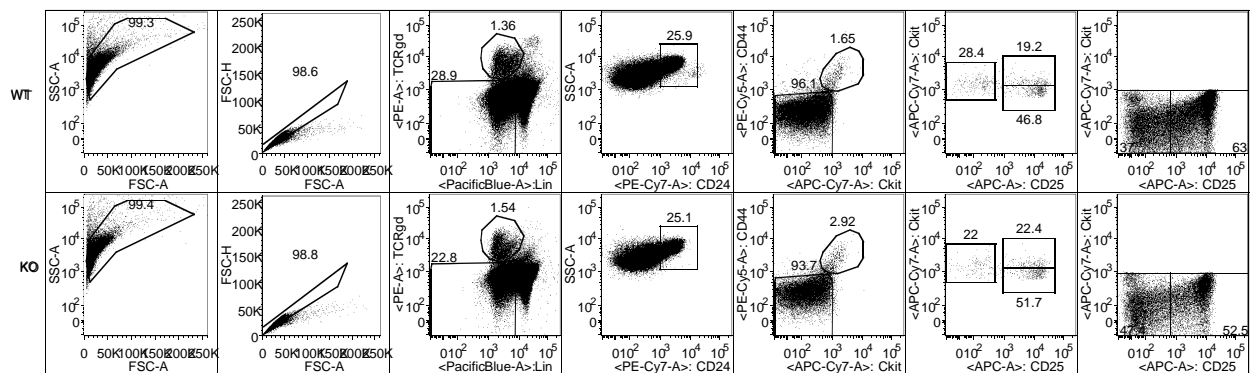

Supplement: S14 Fig — A. Gating strategy for Fig 11A. B. Gating strategy for Fig 11G. (PDF) [file pbio.1002370.s015.pdf]

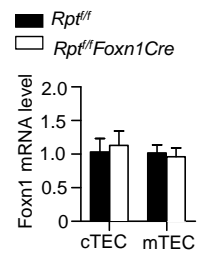

Supplement: S15 Fig — Relative Foxn1 mRNA levels in sorted TECs from 10-d-old mice were determined by real-time qPCR. (PDF) [file pbio.1002370.s016.pdf]

**A**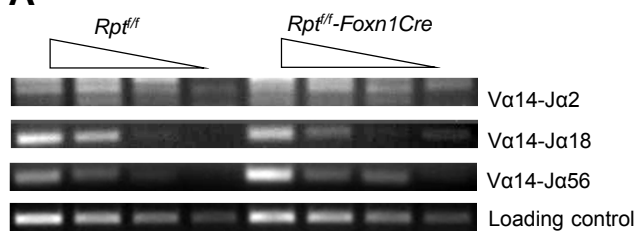**B**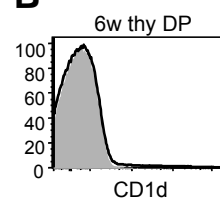

Supplement: S16 Fig — A. Genomic DNA isolated from sorted CD4+CD8+ DP thymocytes from Rpt f/f and Rpt f/f -Foxn1Cre mice were utilized for detection of Vα14 to Jα2, Jα18, and Jα56 recombination using semi-quantitative PCR. TSC1 was used as loading control. B. Overlaid histograms show CD1d expression on DP thymocytes. Data shown represent three experiments. (PDF) [file pbio.1002370.s017.pdf]
