# Supplementary material for: mTORC1 in Thymic Epithelial Cells Is Critical for Thymopoiesis, T-Cell Generation, and Temporal Control of γδT17 Development and TCRγ/δ Recombination
Source: PLoS Biol. 2016 Feb 18;14(2):e1002370. doi: 10.1371/journal.pbio.1002370 (PMC4758703; doi:10.1371/journal.pbio.1002370)
Supplement: S7 Fig — A. Gating strategy for Fig 6A and 6D. B. Gating strategy for Fig 6H. C. Gating strategy for Fig 6I and 6M. (PDF) [file pbio.1002370.s008.pdf]

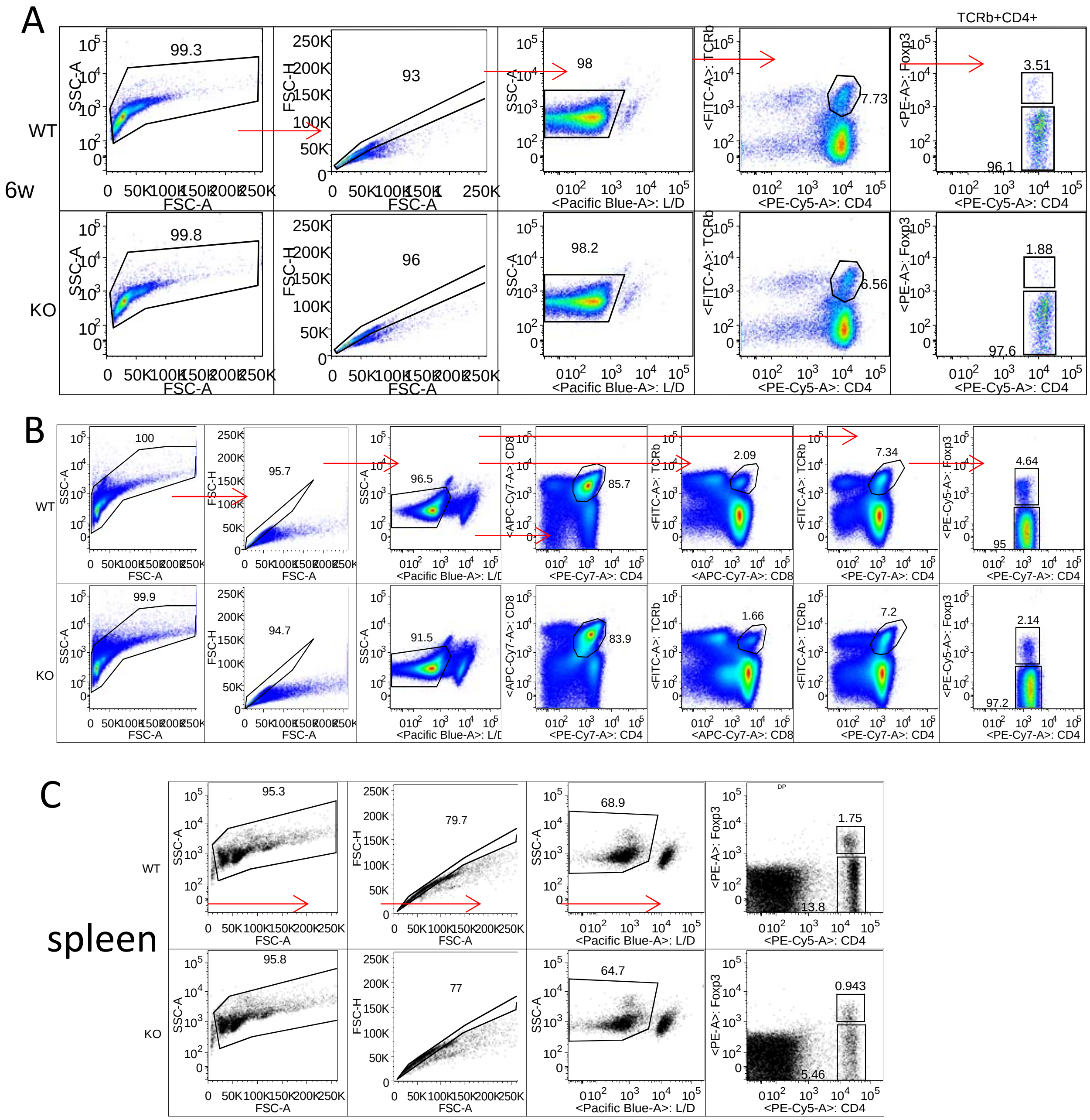
A
6w
WT
KO
TCRb+CD4+
99.3
93
98
7.73
3.51
96.1
99.8
96
98.2
6.56
1.88
97.6
SSC-A
FSC-A
FSC-H
<Pacific Blue-A>: L/D
<FITC-A>: TCRb
<PE-Cy5-A>: CD4
<PE-A>: Foxp3
B
WT
KO
100
95.7
96.5
85.7
2.09
7.34
4.64
95
99.9
94.7
91.5
83.9
1.66
7.2
2.14
97.2
<APC-Cy7-A>: CD8
<PE-Cy7-A>: CD4
<PE-Cy5-A>: Foxp3
C
spleen
WT
KO
95.3
79.7
68.9
1.75
13.8
95.8
77
64.7
0.943
5.46
DP
